# Cerium oxide nanoparticles attenuate hepatic failure via blocking TGF-β/Smads and upregulating Nrf2/HO-1 signaling pathways in liver fibrosis rat model

**DOI:** 10.1007/s00210-025-04435-x

**Published:** 2025-07-18

**Authors:** Noha A. Mowaad, Sara M. Baraka, Saber Ibrahim, Doaa A. Mansour, Reda M. S. Korany, Ahmed F. El-Sayed, Arwa A. Hassan

**Affiliations:** 1 Narcotics, Ergogenics and Poisons Department, National Research Centre, Giza, 12622 Egypt; 2 Chemistry of Natural Compounds Department, National Research Centre, Giza, 12622 Egypt; 3 Packaging Materials Department, National Research Centre, Giza, 12622 Egypt; 4 Nanomaterials Investigation Lab, Central Laboratory Network, National Research Centre, Giza, 12622 Egypt; 5 Department of Biochemistry and Chemistry of Nutrition, Faculty of Veterinary Medicine, University of Sadat City, P.O. Box 32897, Sadat City, Menoufia, Egypt; 6 Pathology Department, Faculty of Veterinary Medicine, Cairo University, Giza, Egypt; 7 Microbial Genetics Department, Biotechnology Research Institute, National Research Centre, Giza, 12622 Egypt; 8 Egypt Center for Research and Regenerative Medicine (ECRRM), Cairo, Egypt; 9 Pharmacology and Toxicology, Ministry of Health & Population, Cairo, Egypt

**Keywords:** Green synthesis, CeO_2_ nanoparticles, TGF-β/Smads, Nrf2, Liver fibrosis, In silico

## Abstract

T his article is aimed at assessing the anti-fibrotic action of plant-based fabricated cerium oxide nanoparticles (CeO_2_NPs). The *Carissa carandas* aerial parts methanolic extract was utilized to prepare CeO_2_NPs, where the dispersal of particle size distribution and zeta potential value was 61.9 nm and − 18.94 mV, respectively, verifying the successful synthesis of CeO_2_NPs. In rats, CeO_2_NPs were intravenously injected at doses of 0.1 and 0.5 mg/kg twice a week for a month, in parallel with thioacetamide (TAA, 200 mg/kg, i.p). Our findings revealed that CeO_2_NPs alleviated the alteration in serum GPT, GOT, ALP, total bilirubin, albumin, and total protein levels induced by TAA. Additionally, the hepatic oxidative insult due to TAA was inferred by elevation in malondialdehyde and reduction of glutathione content, which in combination with downregulation of SIRT1, Nrf2, and HO-1 in hepatic tissues was improved by CeO_2_NPs. A clear repression in hepatic fibrosis was observed in CeO_2_NP-treated groups as demonstrated by significant downregulation in TGF-β1 and p-Smad 2 and 3 proteins and COL1A1, MMP-2, and α-SMA expressions in hepatic tissues. The histopathological outcomes exhibited a considerable accumulation of collagen fibers in hepatocytes in TAA-challenged rats, which was ameliorated by CeO_2_NPs. Also, CeO_2_NPs dampened the death of hepatic cells via blocking caspase 3 activation as indicated by the immunohistochemical results. The in silico investigations documented the powerful binding affinities of TGF-β1, COL1A1, MMP-2, p-Smad2, p-Smad3, SIRT1, Nrf2, and HO-1 proteins with CeO_2_NPs. In conclusion, this study emphasized the efficacy of CeO_2_NPs as a potent anti-fibrotic agent through upregulating SIRT1/Nrf2/HO-1 and downregulating TGF-β1/Smads pathways.

**Supplementary Information:**

The online version contains supplementary material available at 10.1007/s00210-025-04435-x.

## Introduction

One of the main liver issues that progressively worsens to liver cirrhosis and liver failure is liver fibrosis (Godugu et al. 2023). It results from recurrent attacks of liver injury that can be due to viral infections, drug toxicity, alcoholic, and nonalcoholic steatohepatitis (Miao et al. 2020). Per the statistics data reported by the Centers for Disease Control and Prevention in 2017, 4.5 million persons (or 1.8% of the adult population) were diagnosed to have cirrhosis and chronic liver disorders. Incidents of cirrhosis and chronic hepatic dysfunction accounted for 41,473 fatalities (12.8 per 100,000 individuals). The hepato-toxicant, thioacetamide (TAA), is commonly utilized to evoke a reliable, dose‐, and time‐dependent liver fibrosis in rats as that seen in humans (Wallace et al. 2015; Baraka et al. 2020). TAA caused liver fibrosis through its biotransformation, by cytochrome P450 in the liver cells’ microsomes, to highly reactive metabolites that cause inflammation, oxidant/antioxidant imbalance, and hepatic necrosis (Xie et al. 2012)**.**

Free radicals from TAA’s metabolites trigger oxidant/antioxidant imbalance in liver cells, leading to lobular center necrosis and liver injury (Wang et al. 2013). In addition, oxidants and lipid peroxidation compounds cause the emergence of profibrogenic growth factors, cytokines, and prostaglandins. Therefore, reactive oxygen species (ROS) perform a major function in the beginning of fibrosis via the secretion of numerous profibrotic agents (Crosas-Molist and Fabregat 2015). Hepatic stellate cells (HSCs) activate extracellular matrix (ECM) and cause Kupffer cells and inflammatory cells to secrete cytokines or chemokines, which may be triggered in hepatic fibrosis (Friedman 2008). Also, the pathogenic appearance of fibrosis is due to the disruption in the equilibrium of transforming growth factor‐β1 (TGF‐β1) activity (Dooley et al. 2003). TGF-β1 triggers fibroblasts by stimulating HSCs by boosting the TGF-β1/Smad pathway (Feng et al. 2018). It not only stimulates HSC differentiation into myofibroblasts, but it is also considered a major negative regulator of hepatocyte proliferation (Dooley et al. 2003).

Thus, agents that can inhibit TGF-β1 are excellent options for medicines that can prevent the onset of hepatic fibrosis. Because nanoparticles have superior cellular absorption and distribution than conventional chemical medications, their use may introduce potent therapeutic efficacy. The cerium oxide nanoparticles (CeO_2_NPs) are recognized as the most common nanoparticles with powerful activity against inflammation and oxidation (Charbgoo et al. 2017). It has attained great focus in nanotechnology because of its useful utilization as catalysts, fuel cells, and antioxidants in biological systems (Beaudoux et al. 2016).

Nanoceria can be synthesized through multiple techniques. Compared with biological procedures, chemical and physical approaches are more efficient and have faster production times. However, these techniques have many drawbacks, e.g., the use of dangerous chemicals that can produce toxic waste, while the physical methods are energy-intensive, costly, and environmentally destructive. On the other hand, green synthesis has attained a great interest as an ecofriendly and safe method for the creation of nanomaterials, involving nanoceria. It is also reported to improve biocompatibility compared to the physically or chemically created ones (Dhall and Self 2018). The spectroscopic, microscopic, X-ray, and thermal stability methods are tools that could characterize nanocerium (Hartati et al. 2021).

Chemically, CeO_2_NPs have exhibited powerful antioxidant impacts because of their specific elemental characteristics, which aid in the management of some disorders (Das et al. 2013). Molina et al. have discovered that CeO_2_NPs accumulate mainly in the liver in vivo (Molina et al. 2014). This spontaneous buildup with the great redox action of CeO_2_ offers some untapped medicinal promise for treating liver diseases. The high redox abilities of CeO_2_NPs have been utilized in multiple investigations to alleviate non-alcoholic fatty liver disease (Carvajal et al. 2019). Godugu et al. confirmed the protective impacts of CeO_2_NPs versus bile duct ligation-induced liver fibrosis (Godugu et al. 2023). Furthermore, it has been reported that CeO_2_NPs inhibited TGF-β pathway in the cultured HSCs through minimizing oxidant/antioxidant imbalance (Boey et al. 2021).

Based on our previous results (Baraka et al. 2023) which indicate the neuroprotective ability of green-synthesized CeO_2_NPs against TAA-induced hepatic encephalopathy in rats, the purpose of this study was to elucidate and investigate the possible mode of action of CeO_2_NPs as a hepatoprotective agent. To establish this model, TAA was used to induce liver fibrosis in rats, and CeO_2_NPs were created via the green method by the 70% methanolic extract of *Carissa carandas* aerial parts, as in our previous study (Baraka et al. 2023). Diverse phytochemical components involving flavonoids, terpenes, and simple phenolic constituents that possess potent antioxidant properties have been recognized in the *C. carandas* aerial parts alcoholic extract (Baraka et al. 2023). In addition, biochemical, molecular, histopathological, and immunohistochemical assays were conducted to speculate the precise mechanism of CeO_2_NPs as an intervention protocol for liver fibrosis. As an advanced technique, the in silico studies were accomplished to validate the powerful interactions of CeO_2_NPs with fibrotic-mediators target proteins and antioxidant signaling agents.

## Materials and methods

### Plant material preparation

El-Orman Garden (Giza, Egypt) is where the aerial parts of *C. carandas* were collected. The plant identification was kindly confirmed by eminent botanist Dr. Mohamed Gibali. A voucher sample was stored at the National Research Centre Herbarium. The aerial sections that had been air-dried and ground into a powder were extracted multiple times at room temperature using 70% methanol, as explained by a prior study (Basta et al. 2020). The resultant extract was concentrated and evaporated under low pressure until it was completely dry.

### Preparation of CeO_2_NPs

Within a 150 mL flask with a flat bottom, 25 mL of the plant extract was added to 0.93 g of white CeCl_3_ (3.75 mmol/L, Sigma, Germany) powder. This combination, kept at 50 ± 2 °C, was continuously stirred for 5 to 6 h. Stirring caused the hue to change from a white precipitate to a yellowish brown. After that, the precipitate was heated up for 3 to 4 h at 550 °C to create CeO_2_ nanopowder. Before being administered in vivo, CeO_2_NPs were freshly prepared in Millipore water, followed by a sonication step to prepare a suspension using a Sonicator (Sonics, Vibra-CellTM) set at 230 V for 2 min.

### Biological evaluation

#### Animals and ethics

We utilized 40 mature male Wistar rats that ranged in weight from 180 to 200 g. The National Research Center’s Animal House (Dokki, Giza, Egypt) is where they were purchased. In a temperature-controlled environment with a 12-h light/dark cycle at a temperature of 22 ± 3 °C, they were kept in plastic cages and given regular rat feed pellets (21% protein, 3.48% fat, 3.71% fiber, 0.92% calcium, 0.43% phosphorus, and 2450 kcal/kg energy) supplied by local manufacturer (National Research Centre’s Animal House Colony, Dokki, Giza, Egypt) and water as needed.

The animal study, including the justification for the use of animals, feeding, housing, environmental circumstances, and strategies applied to reduce animal suffering, as well as the anesthesia and euthanasia protocol, was certified by the Institutional Animal Care and Use Committee of the University of Sadat City, Faculty of Veterinary Medicine (Egypt) (Approval No: VUSC-023–1-24). The committee gave its approval to the animal research, attesting to their compliance with the ARRIVE criteria and the Guide for Care and Use of Laboratory Animals published by the United States National Institutes of Health.

#### Drugs and chemicals

TAA (Sigma-Aldrich, St. Louis, USA) was dissolved in sterile saline at a concentration of 0.5 mL/100 g rat body weight for intraperitoneal injection. The quality of all other compounds and solvents was exceptionally high for analysis.

#### Rational and dose selection of CeO_2_NPs

Based on prior research (Manne et al. 2017; Córdoba-Jover et al. 2019; Baraka et al. 2023), two doses of CeO_2_NPs (0.1 and 0.5 mg/kg bw, i.v.) have been selected for the in vivo experiment. These studies certified the safety and therapeutic efficacy of those doses to treat various hepatic disorders in experimental rats.

#### Experimental design and treatment protocol

For this experiment, a random selection of eight rats per group was utilized. Control group: normal rats. CeO_2_NPs 0.5 group: for 4 weeks, rats were given CeO_2_NPs (0.5 mg/kg, i.v.) two times per week. TAA group: TAA (200 mg/kg, i.p.) was given to rats twice a week for 4 weeks (Mansour et al. 2017). CeO_2_NPs 0.1 + TAA and CeO_2_NPs 0.5 + TAA groups: rats received intravenous injections of CeO_2_NPs at 0.1 mg/kg (Córdoba-Jover et al. 2019) and 0.5 mg/kg (Manne et al. 2017), respectively, along with receiving TAA as in the second group at a time interval of 1 h. Medical support (0.5 mL/200 g rat body weight) composed of lactate Ringer and glucose 10% in a ratio 1v:1v was given subcutaneously after TAA injection to avoid the decrease in blood glucose level and kidney dysfunction (Gammal et al. 1990).

After 48 h of the last medication dose, the animals were anesthetized using isoflurane inhalation. The blood samples were collected by heart puncture and separated using a cooling centrifuge (Laben Zentrifugen, 2K15, Sigma, Germany) running for 15 min at 3000 rpm. The collected serum samples were stored at − −80 °C for assessing liver function biomarkers. Following autopsy, the liver was quickly taken from the cadaver, wiped on filter paper, weighed, and then washed with ice-cold saline to remove any residual blood. For further biochemical investigation, a section of the liver tissue was preserved at − −80 °C. The fragments were retained in 10% buffered formalin for histological and immunohistochemical analyses.

#### Biochemical assessments

##### Serum hepatic toxicity biomarkers

The level of total bilirubin (Walter and Gerade 1970) and the concentrations of GOT, GPT, and ALP (glutamic oxaloacetic transaminase, glutamic pyruvic transaminase, and alkaline phosphatase, respectively) were colorimetrically calculated in serum samples (Tietz 1995). According to the method of Burtis et al. (Burtis et al. 1999), the concentrations of serum albumin and total protein were assessed. Commercial diagnostic kits (Spinreact, Gerona, Spain) were used to measure these variables by following the manufacturer’s instructions.

##### Production of tissue homogenate

After thoroughly cleaning the hepatic samples, they were gently rubbed between the filter paper folds and then rinsed with ice water. A 10% homogenate was produced in a 0.05 M phosphate buffer (pH 7) using a Polytron homogenizer. The resultant homogenate was centrifuged for 20 min at 10,000 rpm and 4 °C to remove intact cells, nuclei, erythrocytes, and mitochondria as well as cell debris. The supernatant (cytoplasmic extract) was used for assessing heme oxygenase-1 (HO-1) applying a rat ELISA kit (Cat. No. E0676Ra, Bioassay Technology Laboratory, Shanghai, China) in compliance with the manufacturer’s instructions, as well as TGF-β1 using a rat ELISA kit supplied by Cloud-Clone Corp Technology Co., Ltd., Wuhan, China. To assess nuclear Nrf2, the nuclear fraction was made by re-immersing the nuclear pellet in 500 μL of Nuclear Lysis Buffer. Using a cooling centrifuge, the resulting suspension was spun at 14,000 rpm for 10 min. Using a rat ELISA kit (Northwest Life Science Specialities, LLS, NE 94th Avenue, Suite 201, Vancouver, WA 98662, USA), the resulting supernatant (Nuclear fraction) was applied for nuclear Nrf2 measurement.

##### Liver oxidative stress indicator evaluation

In the liver tissue homogenate, the thiobarbituric acid (TBA) reactive substances assay was used to evaluate the level of MDA; malondialdehyde (Ohkawa et al. 1979). Briefly, lipid peroxidation products were estimated by the determination of the level of TBA reactive substances, which were measured as MDA. The latter is the decomposition product of the process of lipid peroxidation. The principle of the assay depends on the colorimetric determination of a pink pigment product resulting from the reaction of TBA reactive substances with MDA in an acidic medium at high temperature. The resulting pink colored chromogen was measured at 532 nm.

The content of GSH (reduced glutathione) was determined in liver tissue homogenate, as earlier described (Beutler 1963). This method depends on the reaction of SH group in GSH with Ellman’s reagent [5, 5-dithiobis (2-nitrobenzoic acid)] to form a stable color of 5-mercapto-2-nitrobenzoic acid which can be measured calorimetrically at 412 nm.

##### Western blot analysis

Using the kit of ReadyPrepTM (Cat. No.#163–2086, Bio-Rad Inc.), the protein was extracted from the liver samples. The Bradford Protein Assay Kit (SK3041) was utilized to quantify the total protein content in samples, whereas the kit supplier is Bio Basic Inc. (Markham, Ontario, L3R 8T4, Canada). The extracted protein of each sample (20 μg) was then loaded with an equal volume of 2 × Laemmli sample buffer composed of 10% 2-mercaptoethanol, 0.004% bromophenol blue, 4% SDS, 20% glycerol, and 0.125 M Tris HCl at pH 6.8. To ensure the complete denaturation of protein, each resultant mixture was boiled for 5 min at 95 °C. Using TGX Stain-Free™ FastCast™ Acrylamide Kit (SDS-PAGE, Bio-Rad Laboratories Inc., Cat. No: # 161–0181), polyacrylamide gels were conducted. The gel was arranged in a transfer sandwich in the following order from bottom to top: filter paper, PVDF membrane, gel, and filter paper. The sandwich was put in the transfer tank containing 1 × transfer buffer, made up of 25 mM Tris, 20% methanol, and 190 mM glycine. The blot was then run for 7 min at a voltage of 25 V using the Bio-Rad Trans-Blot Turbo, which facilitated the transfer of protein bands from the gel to the membrane. Proteins were isolated by gel electrophoresis and subsequently blotted on nitrocellulose membranes, followed by incubation in a tris-buffered saline composed of Tween 20 (TBST) and 3% bovine serum albumin (BSA) buffer for 1 h at room temperature. The following ingredients made up the blocking buffer: 150 mM NaCl, BSA, 20 mM Tris pH 7.5, and 0.1% Tween 20. We bought p-Smad2 and p-Smad3 primary antibodies from Cell Signaling Technology, Inc., 3 Trask Lane, Danvers, MA, 01923, USA.

The solution of the primary antibody was incubated for a full night at 4 °C. Three to five TBST rinses were performed on the blot for 5 min. At room temperature, the blotted target protein was incubated in secondary antibody solution (Goat anti-rabbit IgG-HRP 1 mg, Novus Biologicals) for 60 min. For 5 min, the blot was rinsed three to five times with TBST. The Super ECL detection kit (Bio-Rad Laboratories, Inc., Hercules, California, USA) revealed the presence of protein bands. As per the manufacturer’s instructions, the chemiluminescent substrate (Clarity TM Western ECL substrate, Bio-Rad, Cat. No. #170–5060) was applied to the blot. Solutions A (Clarity western luminol/enhancer solution) and B (peroxidase solution) were added in equal volumes. Using image analysis software on the ChemiDoc MP imager, the target proteins’ band intensities were assessed through normalization them against the beta actin (housekeeping protein).

##### Determination of SIRT1, COLA1, and MMP2 gene expression

Following the homogenization of hepatic tissues from each group and extracting total RNA using a kit supplied by ZYMO RESEARCH CORP., USA (catalog number# R2072), the quality and amount of isolated RNA were colorimetrically checked using a spectrophotometer (Beckman dual, USA).

By providing the SuperScript IV One-Step RT-PCR kit from Thermo Fisher Scientific (Waltham, MA, USA, Cat. No. 12594100), the reverse transcription step was done, and then polymerase chain reaction (PCR) was performed. The primers that were used are listed in Table 1. The thermal profile for the 48-well plate StepOne instrument (Applied Biosystem, USA) was as follows: reverse transcription at 45 °C for 10 min, RT inactivation and initial denaturation at 98 °C for 2 min, followed by amplification with 40 cycles of 10 s at 98 °C, 10 s at 55 °C, and 30 s at 72 °C. The data of target genes (Sirtuin 1 (SIRT1), COL1A1, and MMP2) and housekeeping gene (GAPDH; Glyceraldehyde-3-phosphate dehydrogenase) were recorded as cycle threshold (Ct). The 2^−ΔΔCt^ equation was utilized to calculate the fold changes of the genes relative to GAPDH expression.

**Table 1:** Primer sequences used for the RT-PCR

Gene symbol	Forward	Reverse	Gene bank accession
SIRT1	TACCAGAACAGTTTCATAGAGCCATCAA	AATGTAGATGAGGCAGAGGTT	NM_001372090.1
COL1A1	GTACATCAGCCCAAACCCCAAG	CGGAACCTTCGCTTCCATACTC	NM_053304.1
MMP2	ACCTGAACACTTTCTATGGCTG	CTTCCGCATGGTCTCGATG	NM_031054.2
GAPDH	CACCCTGTTGCTGTAGCCATATTC	GACATCAAGAAGGTGGTGAAGCAG	NM_001394060.2

*SIRT1*, Sirtuin 1; *COL1A1*, Collagen 1A1; *MMP2*, Matrix metalloproteinase 2; *GAPDH*, Glyceraldehyde-3-phosphate dehydrogenase

#### Histopathological analyses

After cleaning, dehydrating, paraffin-embedded, 5-μm thickness sectioning, and hematoxylin and eosin staining steps of the preserved hepatic tissues in neutral-buffer formalin, the histopathological analysis was done using an Olympus BX50 light microscope (Japan) (Bancroft, J. D., & Gamble 2008). Some liver sections were stained with Picrosirius red to evaluate liver fibrosis and examined by bright-field and polarized microscopy.

##### Scoring of histopathological lesions

The grading of liver histopathological changes was based on percentages, with < 30% changes being considered mild, < 30%–50% changes being moderate, and > 50% changes being severe. There were no changes (0), mild (1), moderate (2), and severe (3) alterations (Saleh et al. 2022). Additionally, the area% of liver fibroplasia was quantified using Image J 1.52 p software (National Institutes of Health, USA).

##### Immunohistochemistry studies

Immunohistochemical investigations of caspase-3 and α-SMA were carried out as outlined by Shamseldean et al. (Shamseldean et al. 2022). Sections of liver tissue were treated with xylene to remove paraffin and then rehydrated using alcohol of varying concentrations. To inhibit the activity of endogenous peroxidase, Hydrogen Peroxide Block (Thermo Scientific, USA) was incorporated. Antigen retrieval was achieved by pre-treating tissue sections with 10 mM citrate in a microwave oven for 10 min. Sections were incubated for 2 h with one of the following primary antibodies: anti-caspase-3 antibody at a concentration of 1:1000 (ab4051; Abcam, Cambridge, UK) and mouse monoclonal antibody to alpha smooth muscle actin (α-SMA) diluted 1:100 (M0851; mouse anti-SM-α actin, clone, DAKO, Santa Clara, US). The sections were washed with PBS and then incubated with Goat anti-rat IgG H & L (HRP) (ab205718; Abcam, Cambridge, UK) for 10 min. The sections underwent another rinse with PBS. Finally, the sections were incubated with 3, 3′-diaminobenzidine tetrahydrochloride (DAB, Sigma). The slides were mounted after being counterstained with hematoxylin. For the negative controls, PBS was substituted for the primary antibodies. Five liver slices per group were analyzed to examine the immunoreaction of caspase-3 and α-SMA, as previously reported (Madkour et al. 2022). Using the color deconvolution image J 1.52 p software (Wayne Rasband, National Institutes of Health, USA), the percentage of positively stained cells (%) was calculated.

### Molecular docking simulation

To inspect the activity of CeO_2_NPs, all protein receptors were retrieved from the Protein Data Bank (Table 1S, Supplementary file). These targets were chosen to cover all biomarkers. The preparation of structures involved the elimination of water molecules, ions, and existing ligands using PyMOL software. Hydrogen atoms were added using Autodock Vina. These structures were saved in a pdbqt format for subsequent molecular docking. The structure of the CeO_2_NPs was optimized as follows: a Ce_19_O_32_ cluster was used as a model of CeO_2_NPs (Loschen et al. 2008). We used VESTA (Visualization for Electronic STructural Analysis), 3D visualization program for structural models, volumetric data, and crystal morphologies as follows: (1) the coordinate’s xyz format was converted to CIF format (Crystallographic Information File); then, the cif format was converted to 3D Mol2 format and further converted using Open Babel to 3D PDB format. (3) The structure in PDB format was used as input for the docking simulation. (4) Internal degrees of freedom were set to zero. (5) The structure was changed to the pdbqt format using Autodock tools. (6) The chemical structures were subjected to energy minimization. Finally, the resulting pdb format of CeO_2_NPs was utilized as input for the docking, and Gasteiger-type polar hydrogen charges were assigned to the CeO_2_NPs. Then, CeO_2_NPs were converted to the pdbqt format using Autodock tools. Before docking, the Auto Grid generated ligand-centered maps. Also, the Discovery Studio 4.5 was used to analyze the interactions between the target and ligand structures.

### Statistical analyses

The Shapiro–Wilk test for normality indicated that the data followed a normal distribution at *p* > 0.05. Our records displayed as means ± S.E were statistically analyzed utilizing the Statistical Package for Social Science 17.0 (SPSS Inc., Chicago, IL, USA). One-way analysis of variance (ANOVA) protocol was chosen followed by the Tukey’s HSD post hoc test for multiple comparisons at *p*–value less than 0.05 or 0.001.

## Results

### Characterization of CeO_2_NPs

#### X-ray diffraction

**Fig. 1:**
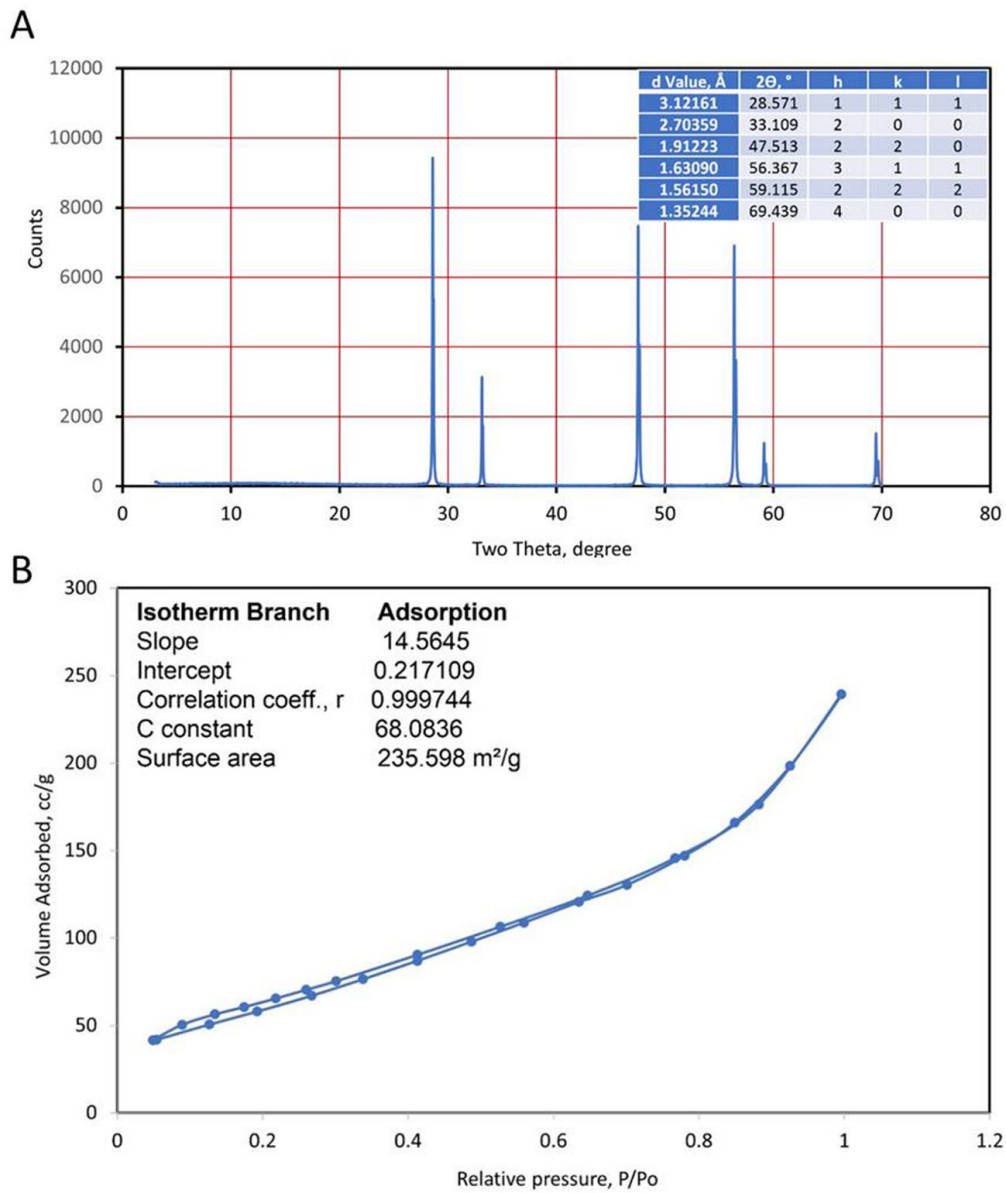
X-ray diffraction patterns (**A**) and complete adsorption–desorption isotherm of nitrogen gas (**B**) of CeO_2_NPs

The XRD bands in Fig. 1A show the characteristic peaks of synthesized CeO_2_NPs. The six main peaks are spotted with defined and identified count values. The 2ϴ of detected peaks can confirm the phase and the typical standards for CeO_2_ lattice as a fingerprint.

The visible peaks illustrated precise 2ϴ values and Miller indices (hkl) as presented in Fig. 1A. The obtainable peaks and noticed values have an excessive identity character with CeO_2_ lattice. Nice slight peaks can also argue to small particle size of synthesized nanomaterial.

#### Surface area

The outer and inner surface area of nanomaterial was determined as the complete-isotherm BET of the CeO_2_ sample (Ibrahim et al. 2021). The contrast relationship between the volume to size of nanomaterial exhibits a nice character with a big surface area. Nitrogen ads.-des. of CeO_2_NPs was in the range from 0.055 to 0.995 P/P_o_ (Fig. 1B). The measured surface area from ads. isotherm for mono-layer adsorbed was 235.6 m^2^/g. The complete isotherm of synthesized CeO_2_ nanomaterial shows a very nice desorption character for nitrogen release as exhibited in the volume adsorbed loop. The results of mean particle size and calculated surface area show a good agreement with each other, as nanomaterial behavior.

#### Particle size and zeta potential

Dynamic light scattering (DLS) is a precise technique to examine the average particle size of suspended nanomaterial (Ibrahim et al. 2022). Additionally, zeta potential as a charge detection value can be used to measure the stability behavior of dispersed nanomaterial (Ibrahim et al. 2019).

**Fig. 2:**
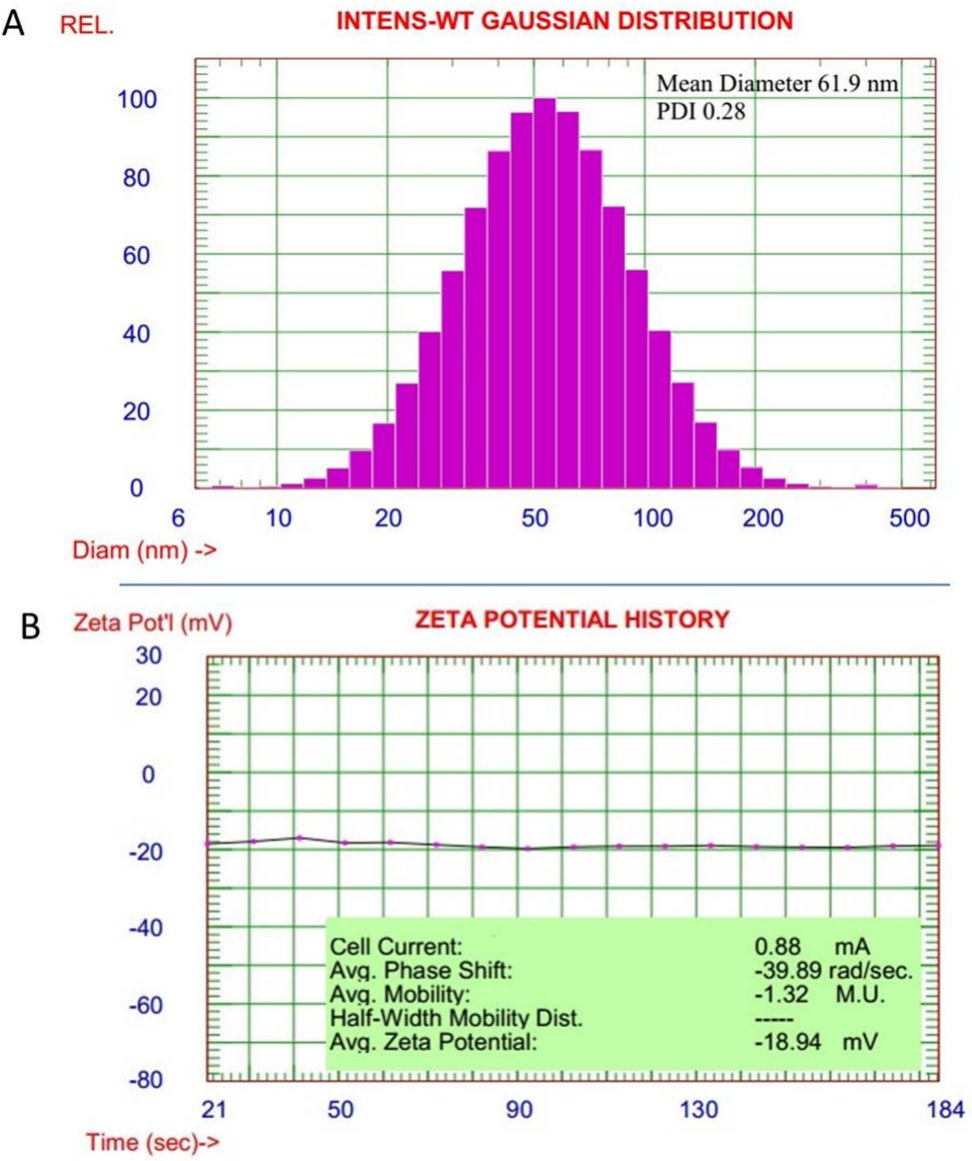
Particle size distribution (**A**) and zeta potential (**B**) of CeO_2_NPs

Figure 2 presents the mean particle size of CeO_2_ and zeta potential series of values. The average radius of the synthesized nanomaterial was 61.9 nm, and polydispersity was 0.28 with a nice uniform particle distribution. In addition, the mean value for zeta potential of different measurements was − 18.94 mV, which pointed to respectable nanomaterial dispersed behavior in Millipore water. Generally, particle size distribution and zeta potential value effectively establish the synthesis of CeO_2_ nanomaterial with good dispersion stability.

### Biochemical analyses

#### Effects of CeO2NPs treatment on liver biomarker indices

The data obtained from Table 2 inferred the hepatic dysfunction in TAA-group as demonstrated by significant rise in GPT (2.66 fold), GOT (1.74 fold), ALP (2.44 fold), and T. bilirubin (1.61 fold) levels coupled with marked decline in T. protein (66.20%) and albumin (62.75%) contents in relative to the control animals. Conversely to that, intravenous injections of CeO_2_NPs at doses of 0.1 or 0.5 mg/kg bw significantly mitigated liver damage as evidenced by preserving liver protein and reducing liver enzymes and T. bilirubin levels when compared to the TAA-hepatotoxic group. It is worth mentioning that rats treated with CeO_2_NPs per se did not record any difference in these hepatic function tests relative to the control group.

**Table 2:** The effects of CeO_2_NP injection on serum hepatic markers in TAA-induced hepatotoxicity rat model

Groups﻿	Control	CeO_2_NPs 0.5	TAA	CeO_2_NPs 0.1 + TAA	CeO_2_NPs 0.5 + TAA
Markers					
GPT (U/L)	38.54 ± 0.88 ^c^	41.62 ± 1.76^c^	102.60 ± 3.89 ^a^	54.51 ± 2.19^b^	43.74 ± 0.77^c^
GOT (U/L)	76.62 ± 2.03^d^	77.08 ± 2.55^d^	133.61 ± 2.25^a^	103.99 ± 3.85^b^	90.36 ± 1.84^c^
ALP (U/L)	200.03 ± 1.75^d^	202.07 ± 1.40^d^	489.07 ± 7.76^a^	415.26 ± 7.85^b^	284.08 ± 10.62^c^
T. protein (g/dL)	9.87 ± 0.27^a^	9.50 ± 0.32^a^	3.34 ± 0.27^b^	7.58 ± 0.39 ^c^	9.24 ± 0.12^a^
Albumin (g/dL)	3.94 ± 0.14^a^	3.64 ± 0.19^a, b^	1.47 ± 0.08^d^	2.59 ± 0.06^c^	3.20 ± 0.12^b^
T. bilirubin (g/dL)	2.06 ± 0.06^c^	2.19 ± 0.08^c^	3.32 ± 0.07^a^	2.65 ± 0.16^b^	2.33 ± 0.07^b, c^

Records are illustrated as mean ± S.E. (*n* = 6). Dissimilar letters mean statistically significant (Tukey’s test). For GPT: a vs. b, c (*p* < 0.001), b vs. c (*p* < 0.01). For GOT: a vs. b, c, d (*p* < 0.001), b vs. c (*p* < 0.01), c vs. d (*p* < 0.01). For ALP: a vs. b, c, d (*p* < 0.001), b vs. c (*p* < 0.001), c vs. d (*p* < 0.001). For T. protein: a vs. b, c (*p* < 0.001), b vs. c (*p* < 0.01). For albumin: a vs. c, d (*p* < 0.001), a vs. b (*p* < 0.01), b vs. c (*p* < 0.01). For T. bilirubin: a vs. b, c (*p* < 0.001), b vs. c (*p* < 0.01)

#### Effects of CeO2NP treatment on hepatic oxidant/antioxida***nt biomarkers***

**Fig. 3:**
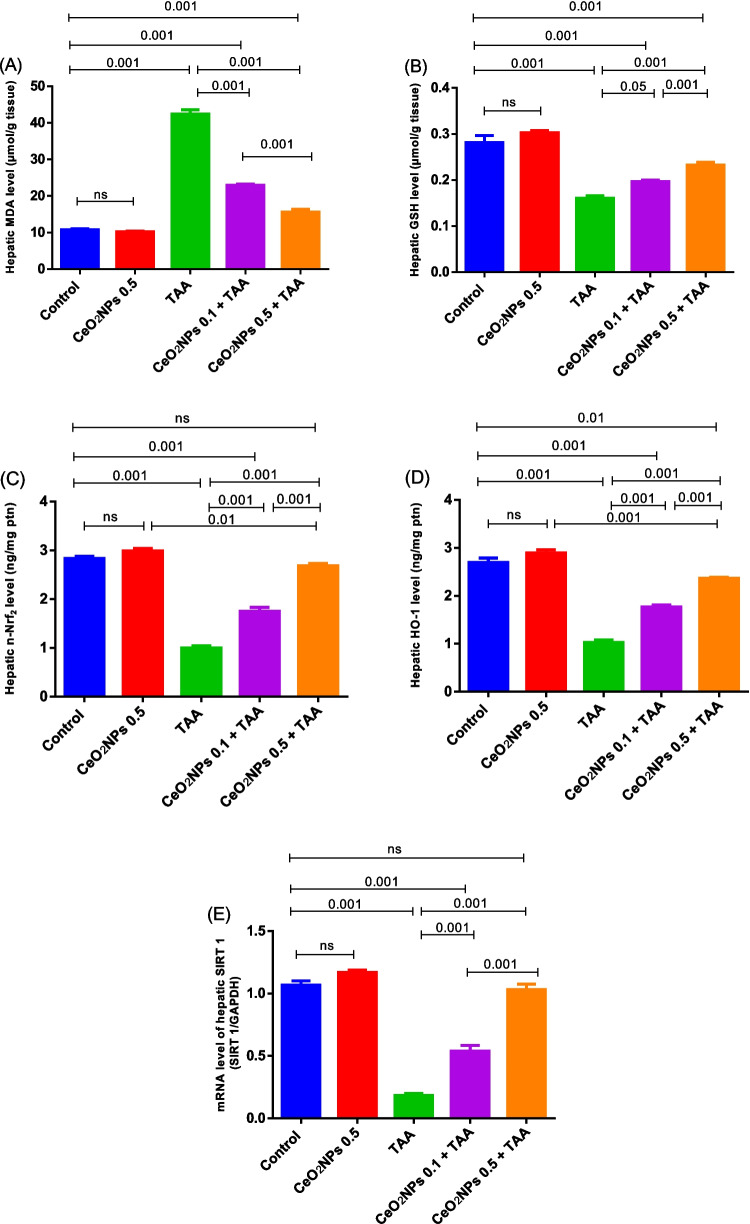
The effects of CeO_2_NP injection on the levels of hepatic MDA (**A**), GSH (**B**), n-Nrf2 (**C**), and HO-1 (**D**), as well as SIRT1 gene expression (**E**), in TAA-induced hepatotoxicity rat model. Each bar represents mean ± SE (*n* = 6). For SIRT 1 gene expression, *n* = 3. ns, non-significant

The incidence of oxidative reactions as response to TA-intoxication was documented by the observed increment in MDA (3.94 fold) level with a depletion of GSH (43.13%) content, in addition to dramatic loss of nuclear Nrf2 and its downstream enzyme, and HO-1 level by 64.69% and 61.93%, respectively, in respect to the normal animals. Moreover, significant downregulation in SRT1 gene expression was depicted in the TAA-model group, compared to the control rats. In contrast, a significant activation of SIRT1/Nrf2/HO-1 signaling pathway, along with repression of MDA content and an increase in GSH level, was reported in the CeO_2_NPs-treated groups, when compared to the TAA-challenged animals. Furthermore, non-significant improvement in these markers was recorded in the CeO_2_NPs 0.5-control group, relative to data of normal rats (Fig. 3).

#### Effects of CeO_2_NPs on hepatic fibrotic markers

**Fig. 4:**
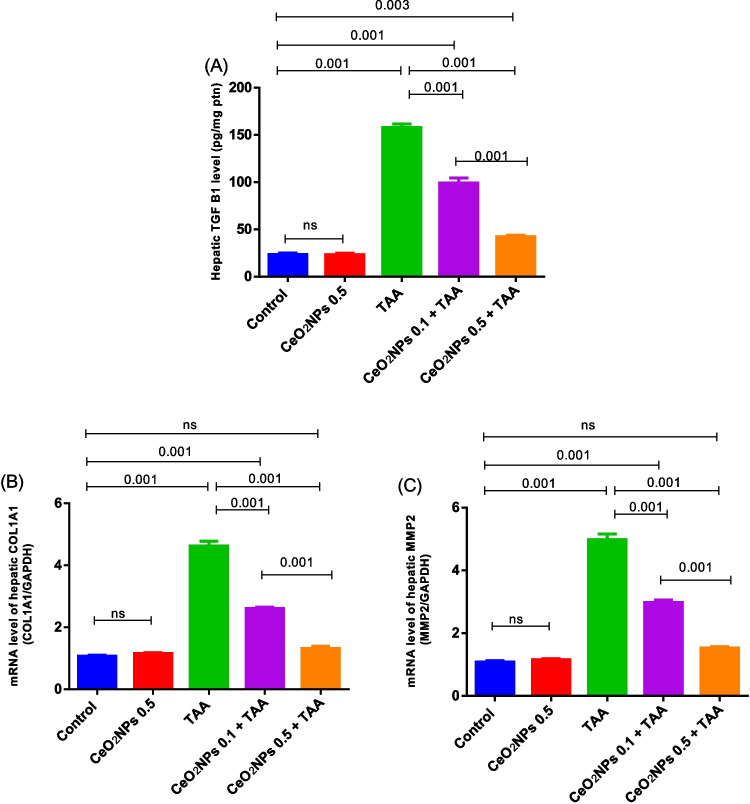
The effects of CeO_2_NP injection on the hepatic TGF-β1 content (**A**), as well as the levels of hepatic COL1A1 (**B**), and MMP2 (**C**) gene expression in TAA-induced hepatotoxicity rat model. Each bar represents mean ± SE (*n* = 6). For RT-PCR, *n* = 3. ns, non-significant

**Fig. 5:**
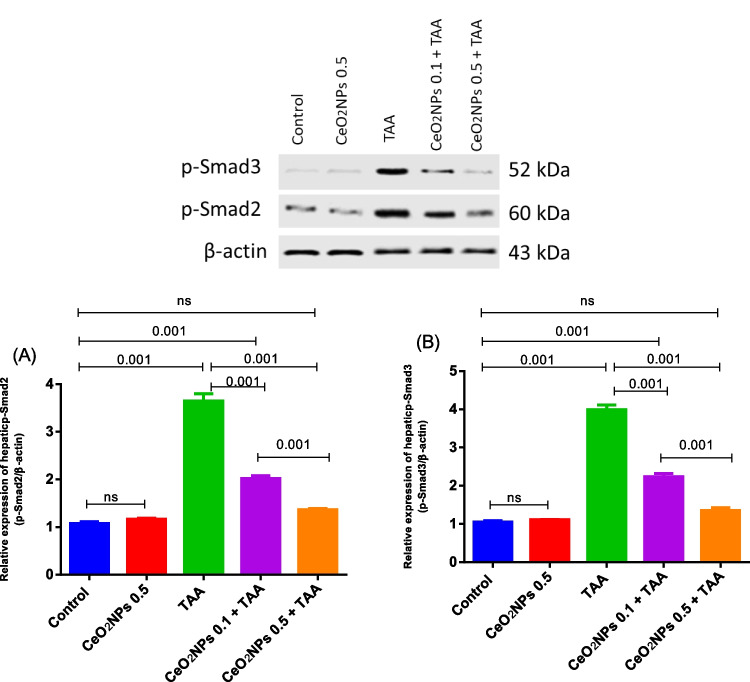
Western blotting analyses of hepatic p-Smad2 (**A**) and p-Smad3 (**B**) in the different groups. Each bar represents mean ± SE (*n* = 3)

As illustrated in Figs. 4 and 5, a remarkable upregulation in TGF-β1 (6.42 fold) level as well as COL1A1 and MMP-2 gene expression, along with a boost in p-Smad2 and p-Smad3 protein expression, was determined in TAA-model group, compared to the normal rats, which reflects the progression of liver fibrosis. On the other hand, rats treated with CeO_2_NPs at the two dose regimens succeeded in suppressing these hepatic fibrotic indicators in a dose-dependent manner, when compared to the TAA-hepatotoxic group. To be noted, intravenous injection of CeO_2_NPs alone demonstrated no changes in the aforementioned parameters relative to the control rats.

### Histopathological findings

#### Hematoxylin and eosin stain

**Fig. 6:**
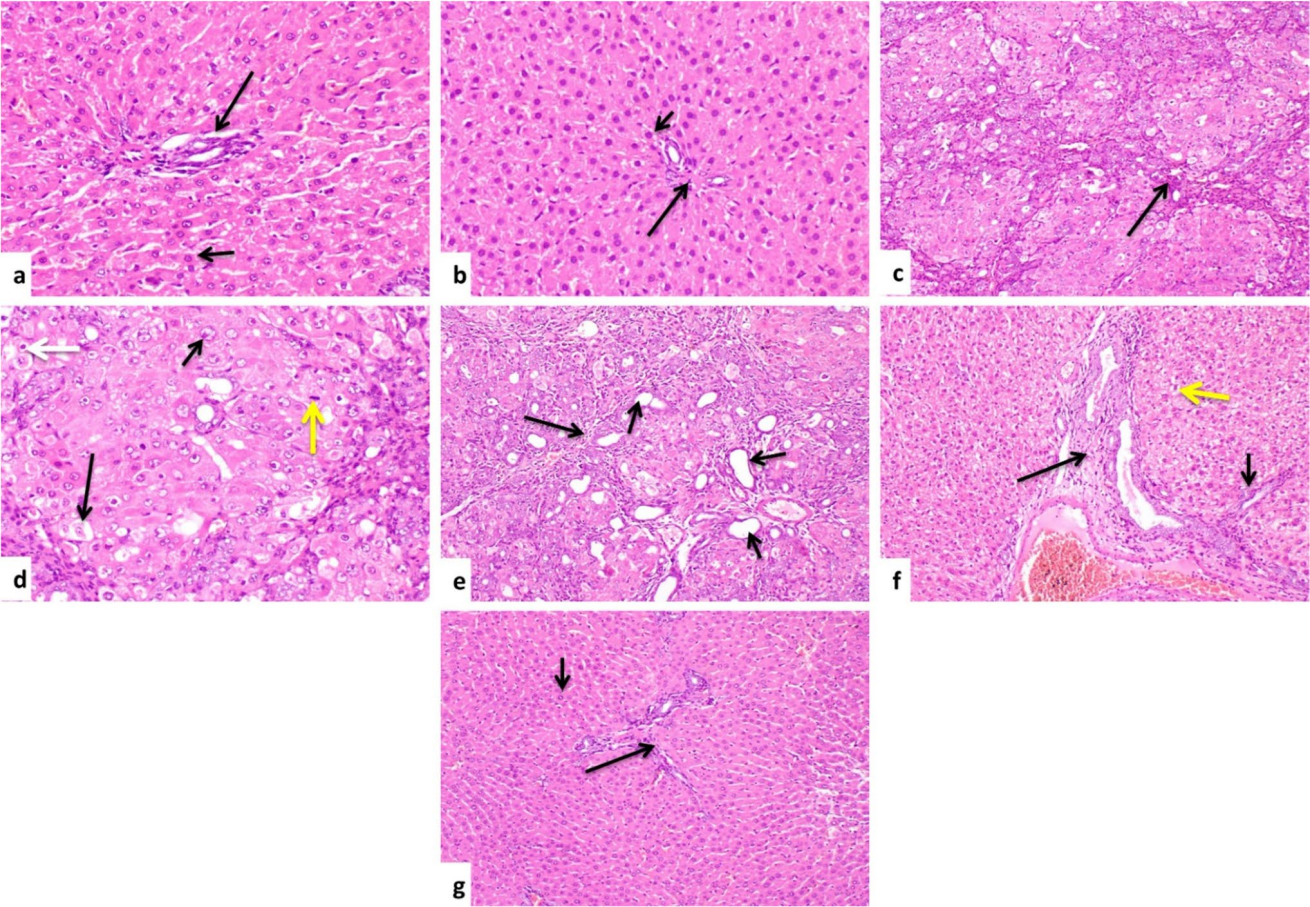
Photomicrograph, liver rat. **a, b** Control and CeO_2_NPs 0.5 groups showing normal structure of portal area (long arrow) and hepatocytes (short arrow) (H&EX200). **c** TAA-intoxicated group showing bridging fibroplasia and pseudolobulation (arrow) (H&EX100). **d** TAA-intoxicated group showing vacuolar degeneration of hepatocytes (long arrow), others showing karyocytomegaly (short arrow), mitotic figures (yellow arrow), and apoptotic bodies (white arrow) (H&EX200). **e** TAA-intoxicated group showing massive portal fibrosis (long arrow), hyperplasia of bile ducts, and formation of newly-formed bile ductules (short arrows) (H&EX100). **f** CeO_2_NPs 0.1 + TAA-treated group showing moderate portal (long arrow) and bridging fibrosis (short arrow) and mild vacuolar degeneration of hepatocytes (yellow arrow) (H&EX100). **g** CeO_2_NPs 0.5 + TAA-treated group showing scanty fibrous tissue between hepatocytes (long arrow) with nearly normal hepatocytes (short arrows) (H&EX100)

Histopathological examination of liver sections from control and CeO_2_NPs 0.5 groups revealed normal structure (Fig. 6a, b). TAA intoxicated group revealed presence of bridging fibroplasia and pseudolobulation of hepatic parenchyma (Fig. 6c) and vacuolar degeneration of some hepatocytes, while others revealed karyocytomegaly; there was also mitotic figures and apoptotic bodies (Fig. 6d); also, this group showed massive portal fibrosis, infiltration of portal areas with mononuclear inflammatory cells, hyperplasia of bile ducts, and formation of newly-formed bile ductules (Fig. 6e). Concerning CeO_2_NPs 0.1 + TAA-treated group, there was improvement in the previous lesions as the portal and bridging fibrosis were moderate with mild vacuolar degeneration of hepatocytes (Fig. 6f). CeO_2_NPs 0.5 + TAA-treated group revealed significant reduction in the aforementioned lesions in form of scanty fibrous tissue between hepatocytes with nearly normal hepatic architecture (Fig. 6g).

#### Scoring of histopathological lesion

Based on the severity of lesions observed in the examined hepatic tissues, a score ranging from 0 to 3 was selected (Table 3).

**Table 3:** Histopathological hepatic alterations scoring

Lesions	Control	CeO_2_NPs 0.5	TAA	CeO_2_NPs 0.1 + TAA	CeO_2_NPs 0.5 + TAA
Bridging fibroplasia Pseudolobulation of hepatic parenchyma Vacuolar degeneration of hepatocytes Karyocytomegaly of hepatocytes Mitotic figures Apoptosis of hepatocytes Portal fibrosis Hyperplasia of bile ducts Formation of newly-formed bile ductules	0 0 0 0 0 0 0 0 0	0 0 0 0 0 0 0 0 0	3 3 3 3 2 2 3 3 3	2 1 2 1 0 1 2 2 1	0 0 1 0 0 1 1 1 1

^*^0, no lesions; 1, lesions less than 30%; 2, lesions range from 30 to 50%; 3, lesions more than 50% (*n* = 5)

#### Histochemical finding

**Fig. 7:**
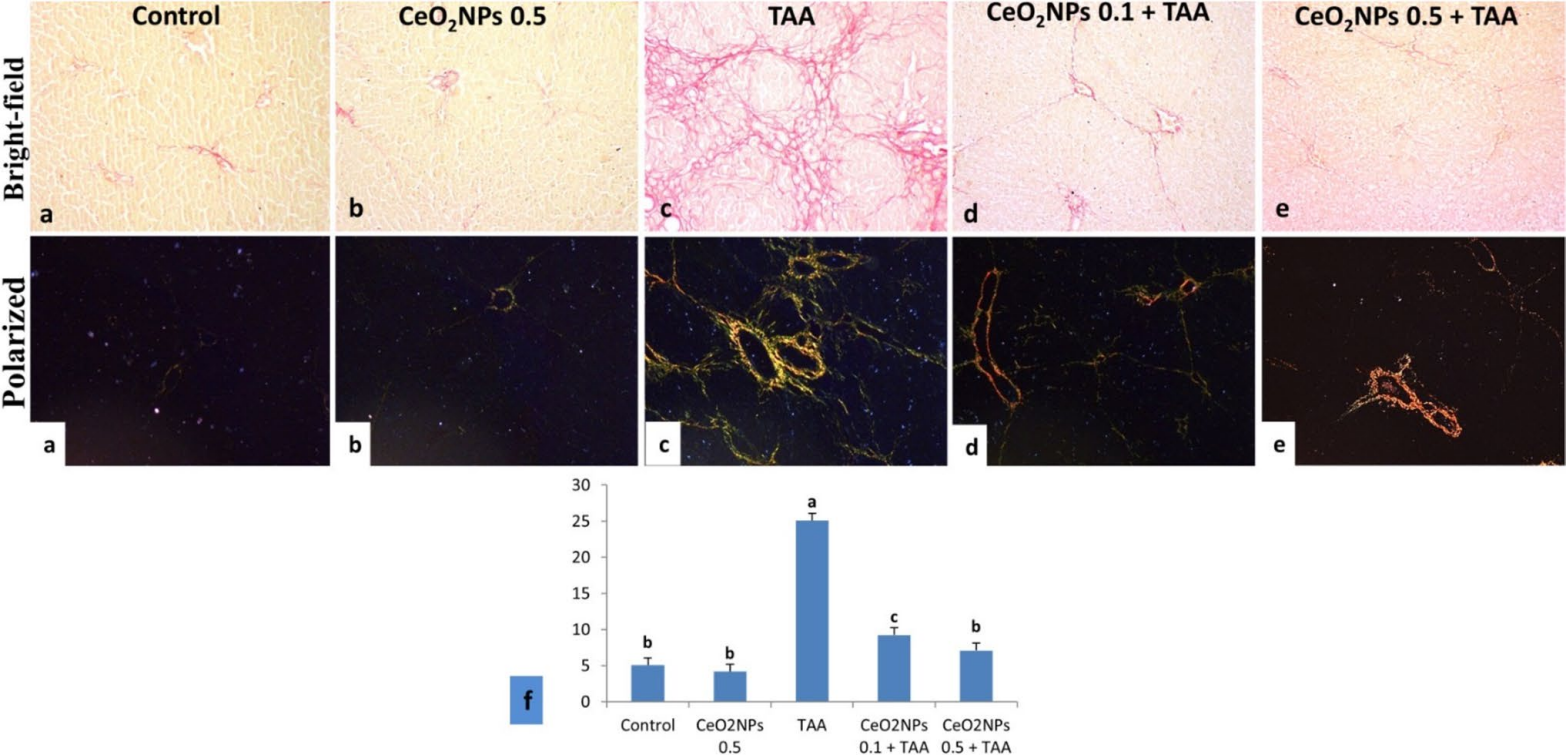
Photomicrograph, bright-field and polarized microscopy of Picrosirius red stained rat liver tissue sections. **a, b** Control and CeO_2_NPs 0.5 groups showing normal deposition of collagen. **c** TAA-intoxicated group showing massive portal and bridging fibrosis. **d** CeO_2_NPs 0.1 + TAA-treated group showing reduced collagen deposition. **e** CeO_2_NPs 0.5 + TAA-treated group, scanty collagen deposition. **f** The percentage area of collagen accumulation, results were illustrated as mean ± S.E (*n* = 5), a vs. b, c (*p* < 0.0001), b vs. c (*p* < 0.001)

Picrosirius red-stained tissue sections were examined by bright field and polarized microscopy for collagen deposition in different experimental groups. Area% of collagen deposition was illustrated in Fig. 7f. The control and CeO_2_NPs 0.5 groups revealed normal deposition of collagen (Fig. 7a, b). TAA-intoxicated group showed massive portal and bridging fibrosis (Fig. 7c), the CeO_2_NPs 0.1 + TAA-treated group revealed noticeable amelioration as the collagen deposition was reduced (Fig. 7d), while in the CeO_2_NPs 0.5 + TAA-treated group, there was a significant reduction in collagen deposition (Fig. 7e).

#### Caspase-3 and α-SMA immunohistochemical studies

**Fig. 8:**
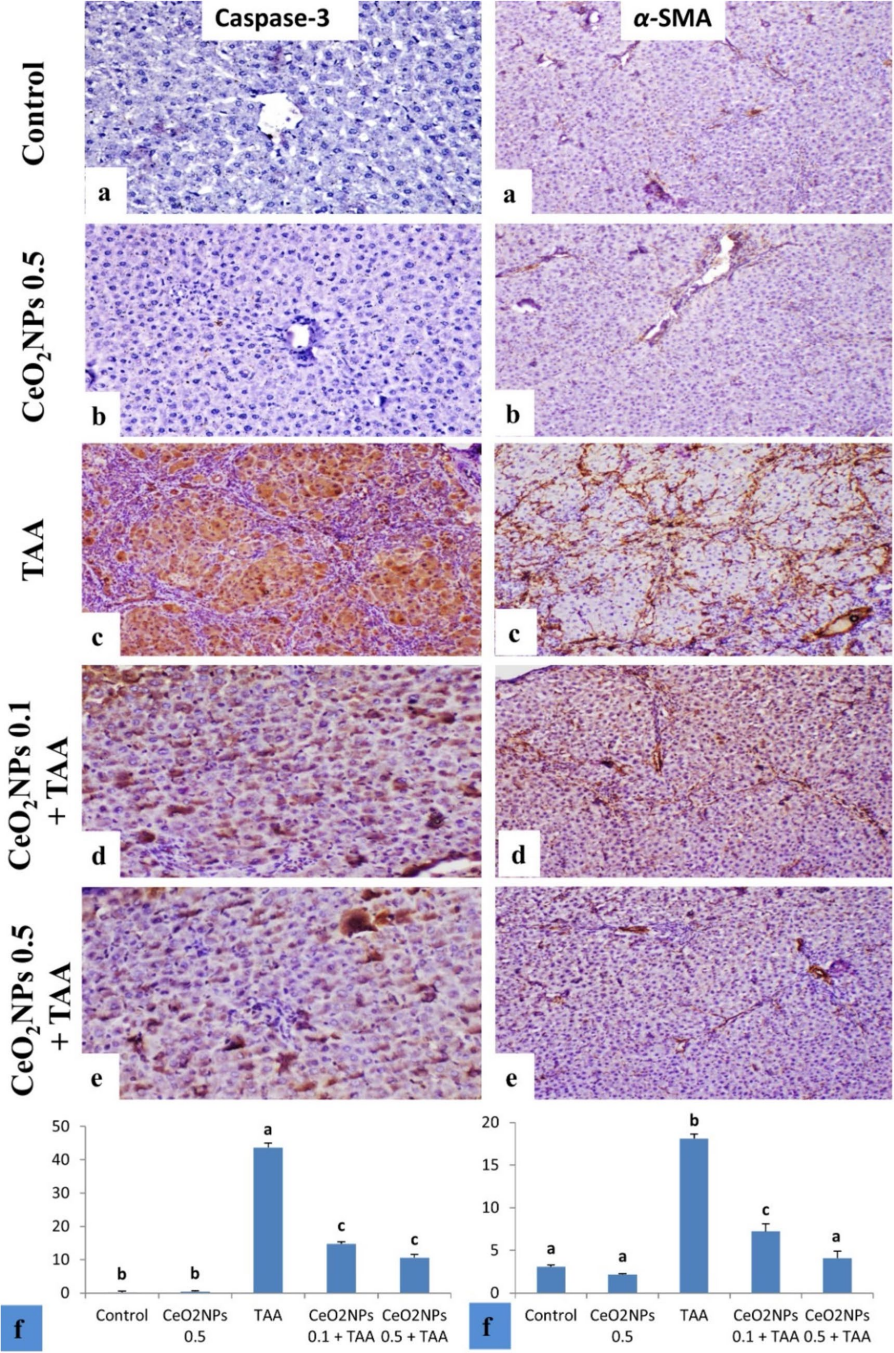
Immunostaining of caspase-3 and *α-*SMA and liver rat. **a, b** Control and CeO_2_NPs 0.5 groups showing immunostaining of caspase-3 in liver with no immune-reactive cells, *α-*SMA in control group and CeO_2_NPs 0.5 treated groups showing expression in smooth muscle wall of blood vessels. **c** TAA-intoxicated group showing strong caspase-3 expression in hepatocytes and strong expression *α-*SMA in vascular walls and also stellate cells in interlobular fibrous septa. **d, e** Groups treated with CeO_2_NPs 0.1 + TAA and CeO_2_NPs 0.5 + TAA, showing weaker positive immune-reaction in fewer hepatocytes, expression of *α-*SMA is reduced in CeO_2_NPs 0.1 + TAA and CeO_2_NPs 0.5 + TAA (caspase-3 and *α-*SMA X 100). **f** Immunostaining expression of caspase-3 and *α-*SMA % area in liver tissues, results were illustrated as mean ± S.E (*n* = 5), a vs. b, c (*p* < 0.0001), b vs. c (*p* < 0.0001)

Immunostaining expression of caspase-3 and *α-*SMA% area in liver tissue of different treated groups was illustrated in Fig. 8f. Immunostaining of caspase-3 in liver revealed no immune-reactive cells in the control group and CeO_2_NPs 0.5-treated groups (Fig. 8a, b). Treated groups with TAA revealed strong expression in hepatocytes (Fig. 8c). Groups treated with CeO_2_NPs 0.1 + TAA and CeO_2_NPs 0.5 + TAA revealed weaker positive immunoreaction in a few hepatocytes (Fig. 8d, e). Concerning *α-*SMA, in control group and CeO_2_NPs 0.5-treated groups showed expression only in smooth muscle wall of blood vessels (Fig. 8a, b), while in TAA-intoxicated group showed strong expression in vascular walls and also stellate cells in interlobular fibrous septa (Fig. 8c), this expression was greatly reduced in CeO_2_NPs 0.1 + TAA and CeO_2_NPs 0.5 + TAA (Fig. 8d, e).

### Docking results

**Fig. 9:**
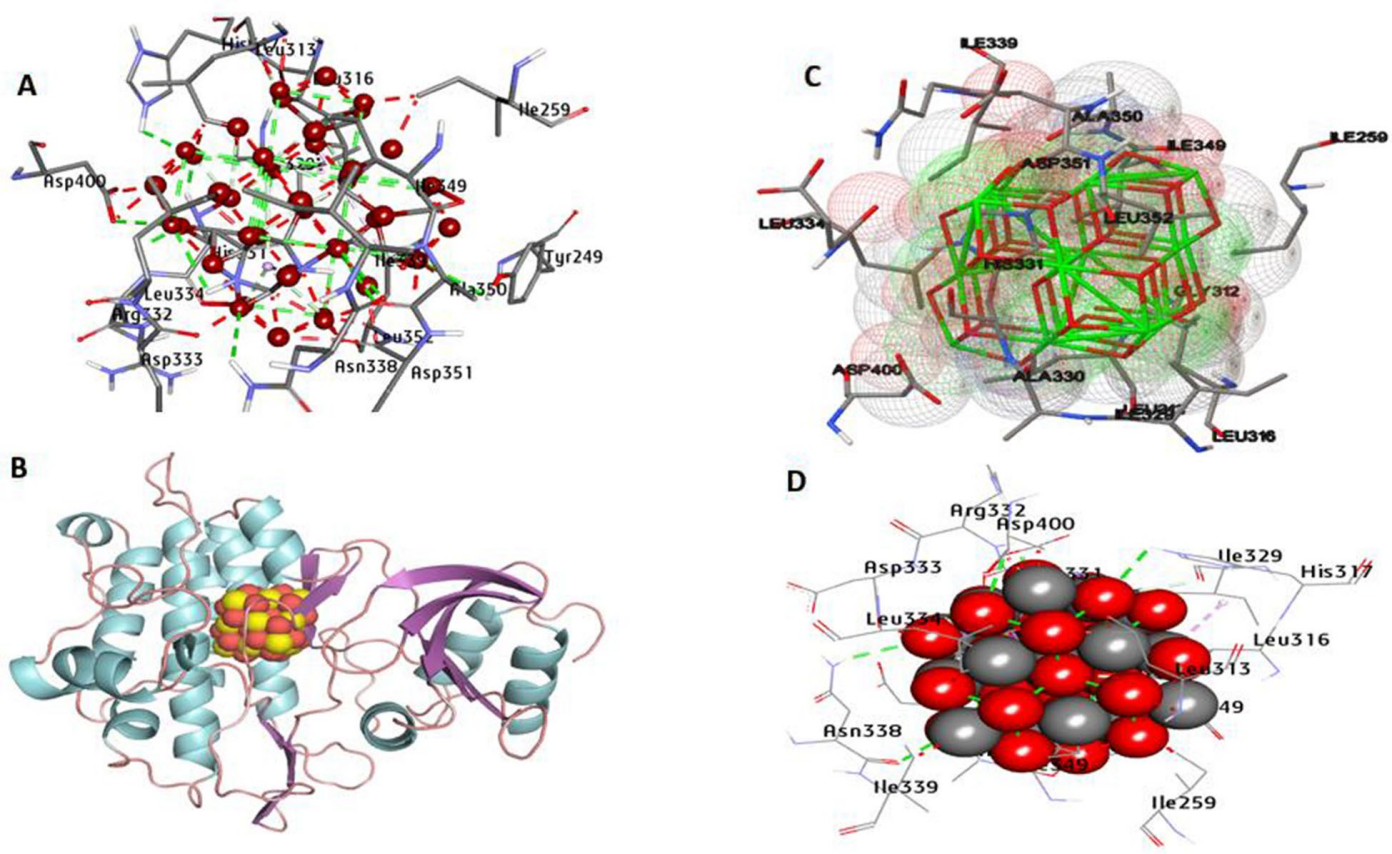
3D depictions of CeO_2_NP conformations within the binding pocket of TGFβ1 proteins (PDB: ID 2X7O). **A** and **D** display the interaction between shared residues and CeO_2_NPs, **B** complex interaction with CNP, and **C** feature interaction with CeO_2_NPs

**Fig. 10:**
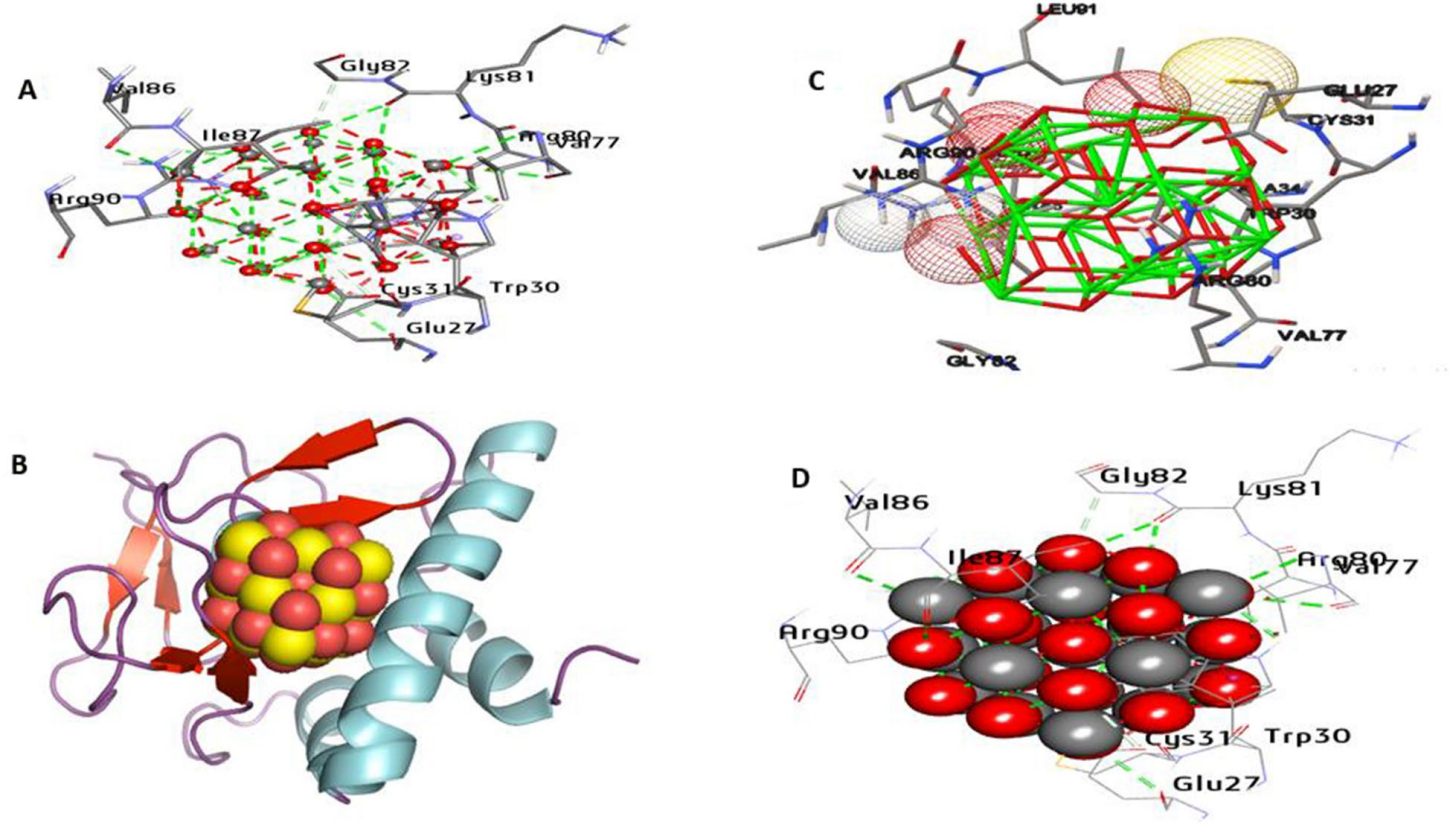
3D depictions of CeO_2_NP conformations within the binding pocket of Smad3 (PDB:ID 1OZJ). **A** and **D** display the interaction between shared residues and CeO_2_NPs, **B** complex interaction with CeO_2_NPs, and **C** feature interaction with CeO_2_NPs

The results of docking analysis are depicted in Table 2S, supplementary file. TGF-β1 is a multifunctional cytokine that regulates cell proliferation, differentiation, immunological activity, and cell integrity. Regulating TGF-β1 signaling is a potential treatment option for some diseases. According to the analysis of docking results, the CeO_2_NPs have an affinity interaction toward TGF-β1 by − 18.55 kcal/mol. CeO_2_NPs formed 12 hydrophilic bonds with the active site of TGF-β1 at Tyr249, His317, His331, Arg332, Asn338, Ala350, and Asp400. In addition, hydrophobic interactions included one Pi-Sigma bond with His331, two Alkyl bonds with Ile329 and Leu352, and six carbon-hydrogen bonds with Leu313, Leu316, and Ile349 have been detected in the activity site. Also, it can be stated that common residues Tyr249, Leu316, Asp400, and Asn338 in the catalytic site enhance the binding affinity (Fig. 9). Smad3 is a key factor in the TGF-β signaling cascade. The docking results between CeO_2_NPs and Smad3 (PDB:1OZJ) are shown in Fig. 10. The predicted binding energies were determined to be − 22.84 kcal/mol. CeO_2_NPs generated eight hydrogen-bonded interactions with Trp30, Val77, Arg80, Arg90, Val86, Ile87, Lys81, and Glu27 in the activity site. Hydrophobic interactions comprised two Pi-Sigma bonds with Trp30 and five carbon-hydrogen bonds with Cys31, Arg80, Gly82, and Trp30. Furthermore, common residues Arg90, Arg80, Trp30, and Val77 in the catalytic site have been shown to increase binding affinity (Fig. 10).

**Fig. 11:**
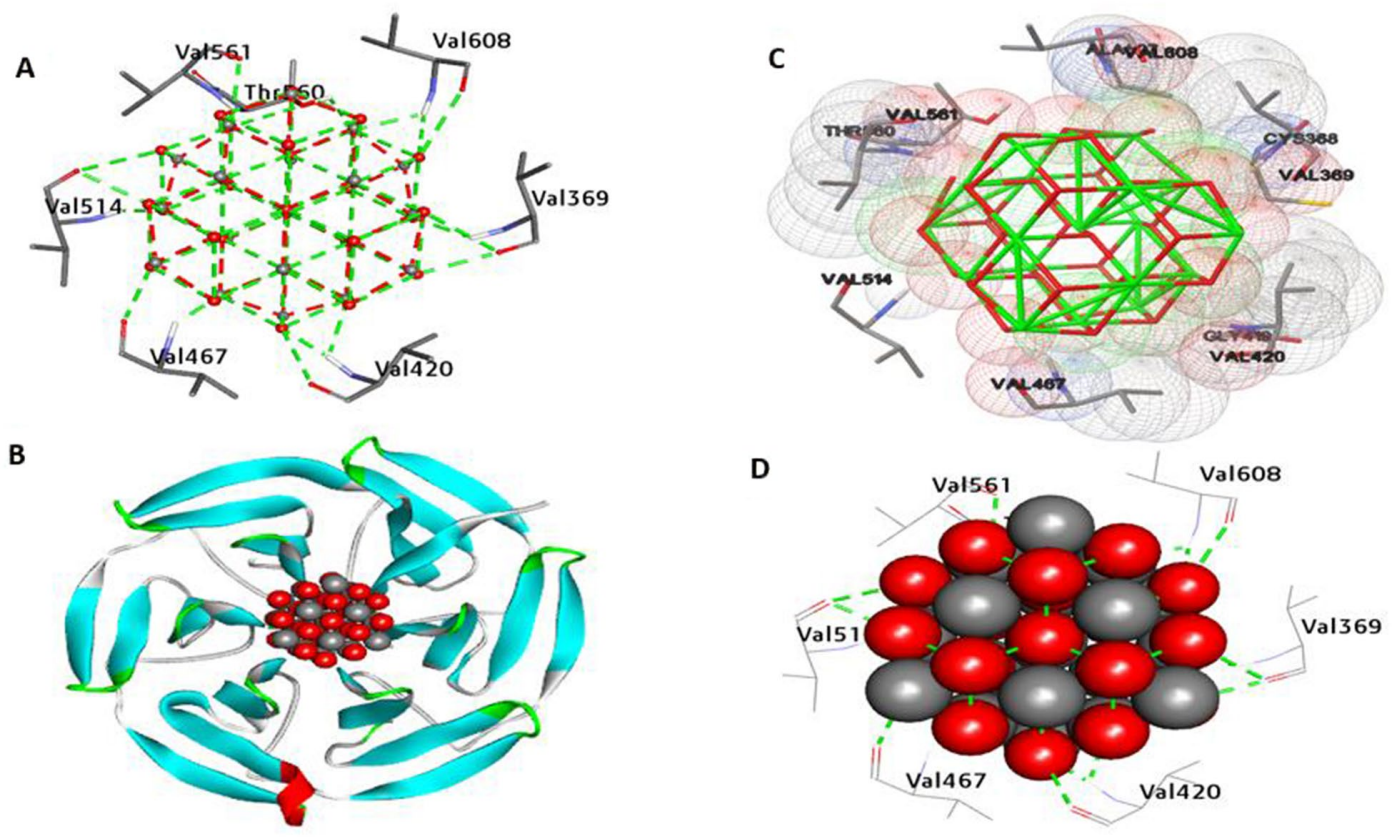
3D depictions of CeO_2_NP conformations within the binding pocket of Nrf2 protein (PDB:ID 7ECA). **A** and **D** display the interaction between shared residues and CeO_2_NPs, **B** complex interaction with CeO_2_NPs, and **C** feature interaction with CeO_2_NPs

Nrf2 is a transcription factor that is essential for biological protection against damage caused by oxidation. The stimulation of Nrf2 is of interest because of potential therapeutic applications in a variety of disorders linked to oxidative stress, inflammation, and pollutant exposure. Docking findings analysis (Fig. 11) shows that CeO_2_NPs have an affinity interaction of − 12.497 kcal/mol. Furthermore, the hydrophilic interaction generated 10 hydrogen bonds with Val369, Val420, Val467, Val514, Thr560, Val561, Val608, and Val369. In addition, two carbon-hydrogen bond interactions were generated within the active pocket with Val608 and Val369. Furthermore, the residues Val369, Val420, and Thr560 in the catalytic site increase binding affinity.

**Fig. 12:**
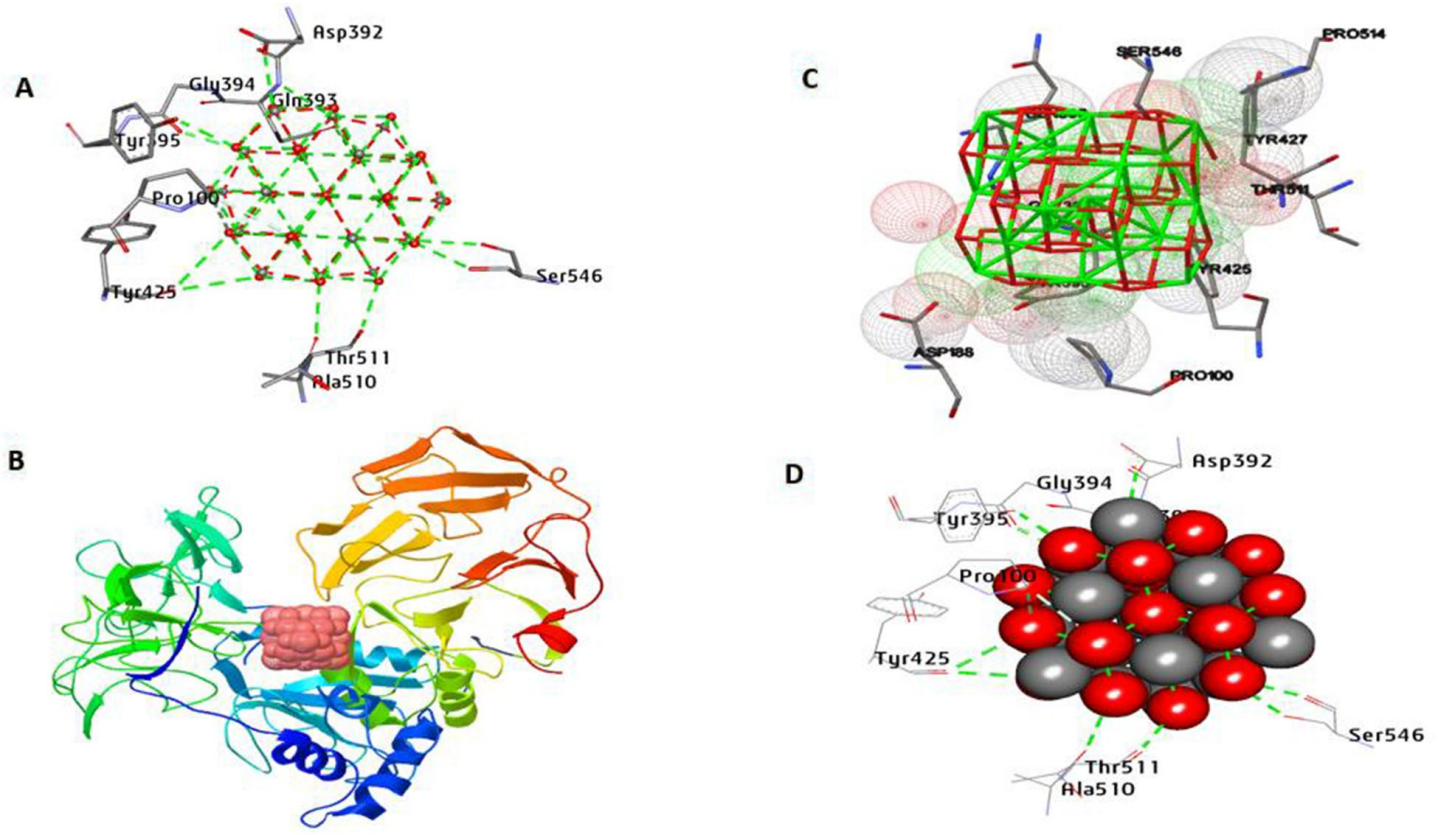
3D depictions of CeO_2_NP conformations within the binding pocket of MMP2 (PDB:ID 1CK7). **A** and **D** display the interaction between shared residues and CeO_2_NPs, **B** complex interaction with CeO_2_NPs, and **C** feature interaction with CeO_2_NPs

By inhibiting MMP2, it is possible to potentially prevent or slow down the progression of certain diseases, particularly those characterized by excessive tissue remodeling or invasive behavior. Docking finding analysis (Fig. 12) shows that CeO_2_NPs have an affinity interaction of − 16.48 kcal/mol. It generated 12 hydrophilic bonds with Gln393, Tyr395, Ser546, Gly394, Tyr425, Asp392, Gln393, Ala510, Ser546, and Thr511. Also, two hydrophobic interactions, including carbon-hydrogen bonds with Pro100, were formed. Moreover, it can be concluded that the amino acids Gln393, Ser546, Ala510, and Asp392 in the activity pocket promote binding affinity.

**Fig. 13:**
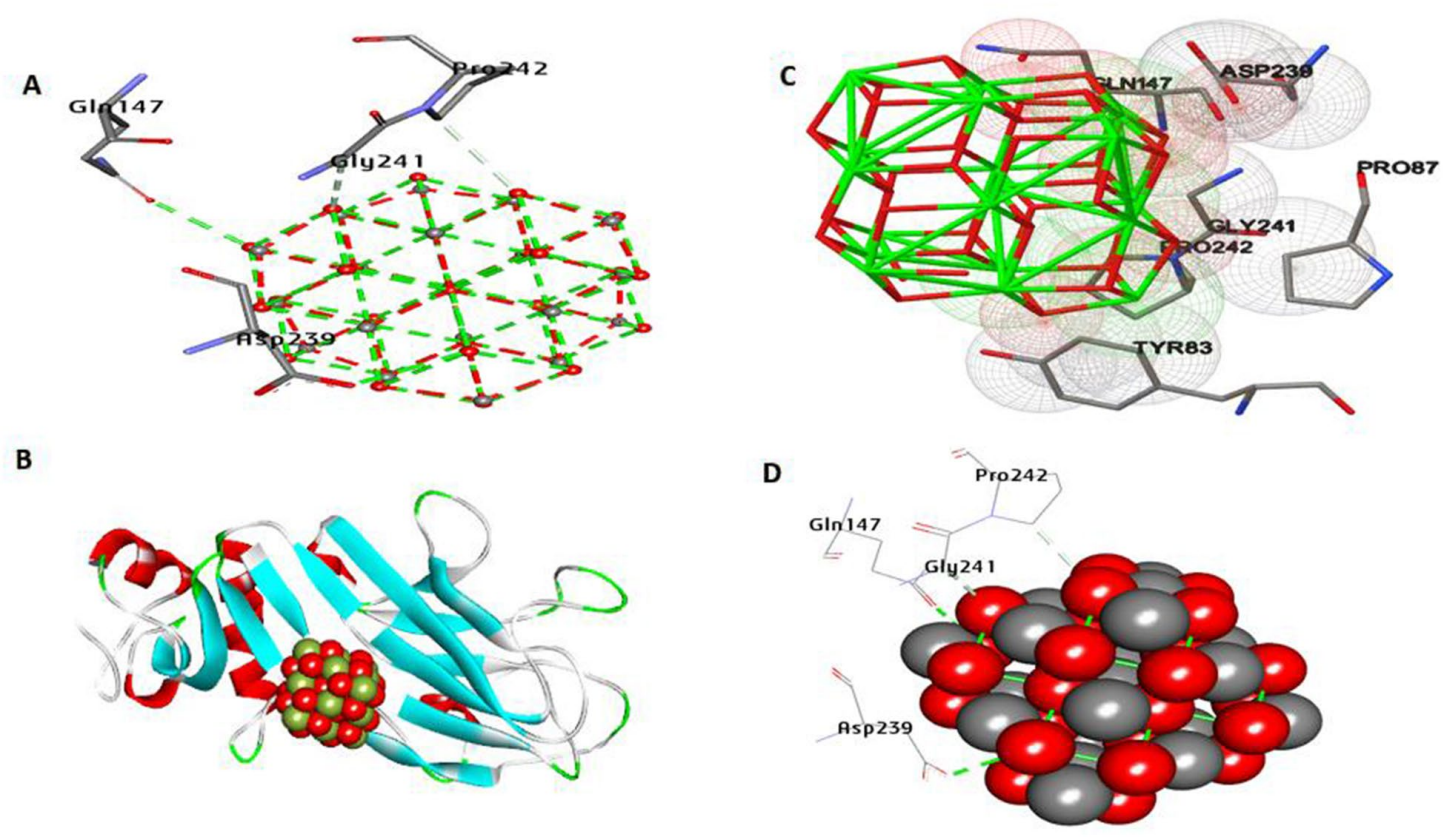
3D depictions of CeO_2_NP conformations within the binding pocket of COL1A1 (PDB:ID 5K31). Interaction between common residues with CeO_2_NPs, **B** complex interaction with CeO_2_NPs, and **C** feature interaction with CeO_2_NPs

COL1A1 is the alpha 1 chain of type I collagen. Docking results (Fig. 13) show that CeO_2_NPs have an affinity interaction of − 6.87 kcal/mol. It made two hydrogen bonds with Asp239 and Gln147. Hydrophobic interactions also resulted in the formation of two carbon-hydrogen bonds with Gly241 and Pro242.

**Fig. 14:**
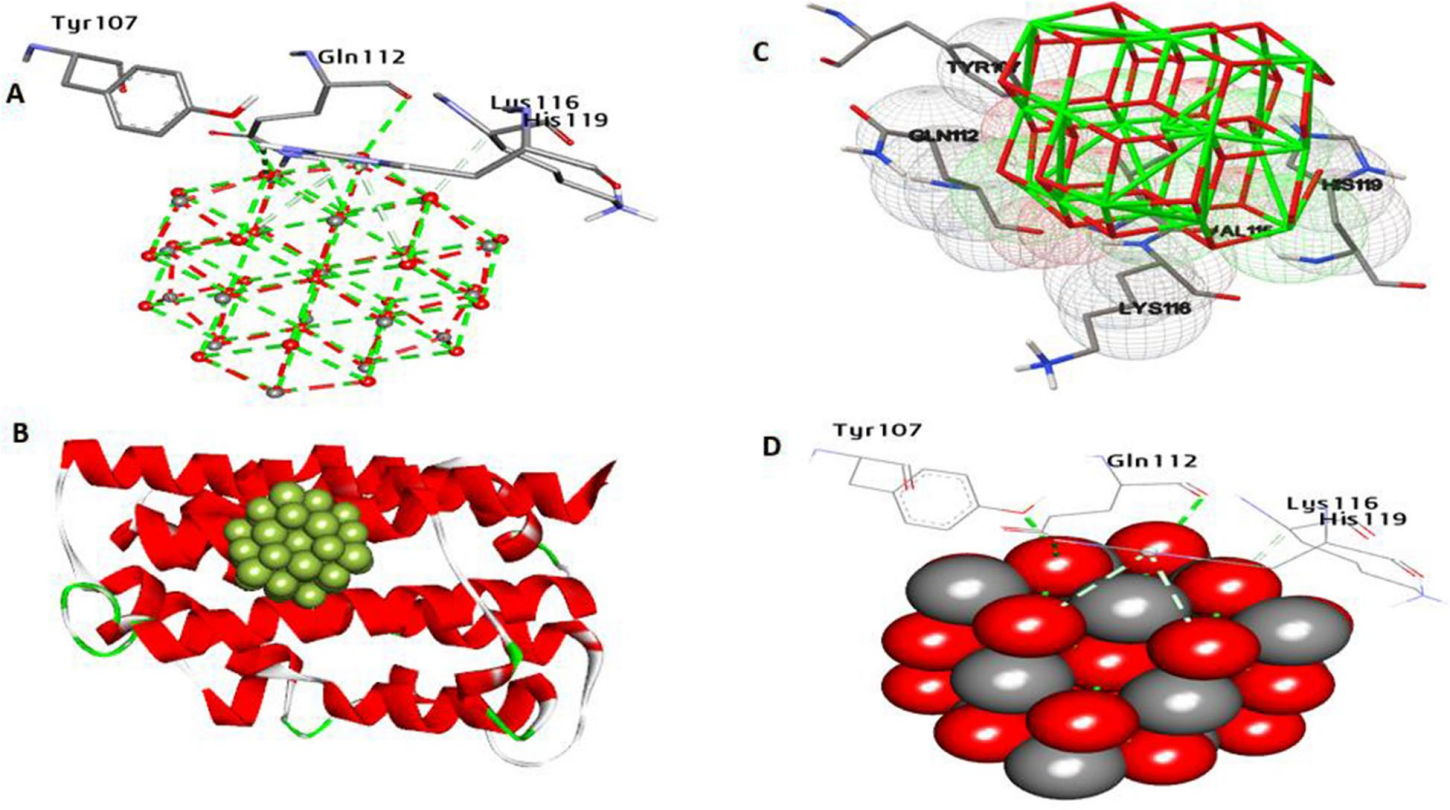
3D depictions of CeO_2_NP conformations within the binding pocket of HO-1 (PDB:ID 1N45). **A** and **D** display the interaction between shared residues and CeO_2_NPs, **B** complex interaction with CeO_2_NPs, and **C** feature interaction with CeO_2_NPs

HO-1 has been found to possess important cytoprotective and anti-inflammatory properties. The control of HO-1 has been linked to a variety of physiological and pathological conditions, including inflammation, oxidative stress disorders, and cancer. Docking data (Fig. 14) show that CeO_2_NPs have an affinity interaction of − 18.10 kcal/mol. It created four hydrogen bonds with Gln112, His119, Gln112, and Tyr107. In addition, two non-hydrophilic contacts within the activity pocket were formed: a carbon-hydrogen bond with Lys116 and a Pi-donor bond with His119. Furthermore, the amino acids Arg152, Thr159, and Gln156 at the catalytic site increase binding affinity.

**Fig. 15:**
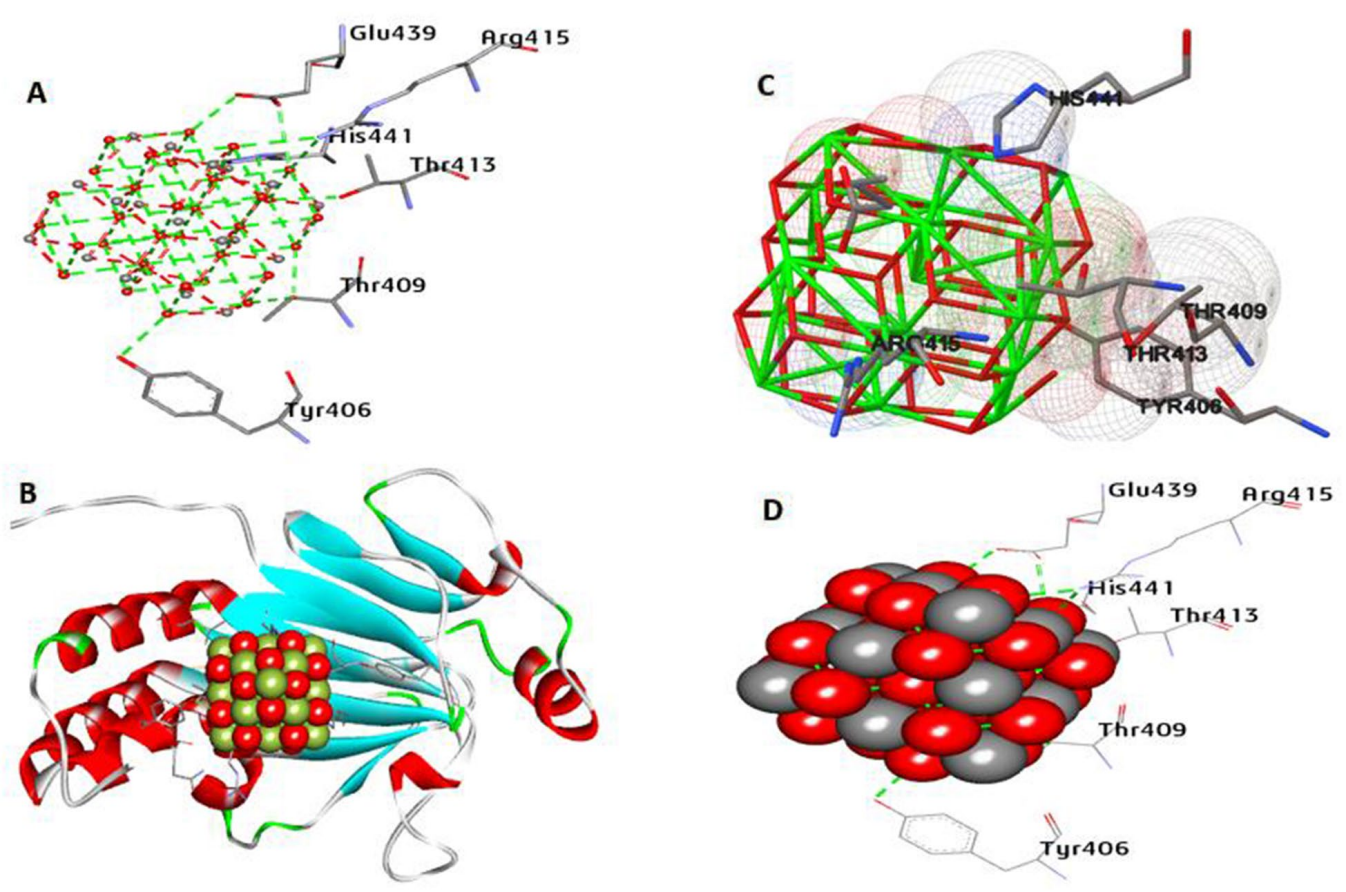
3D depictions of CeO_2_NP conformations within the binding pocket of Smad2 (PDB:ID 1KHX). **A** and **D** display the interaction between shared residues and CeO_2_NPs, **B** complex interaction with CeO_2_NPs, and **C** feature interaction with CeO_2_NPs

Smad2 is a member of the Smad family of transmission of signal proteins. It is essential for transferring signals from the cell surface to the nucleus, where they modulate the expression of genes and impact a variety of cellular processes such as growth, differentiation, and tumorigenesis. The docking results between CeO_2_NPs and Smad2 (PDB: 1KHX) are illustrated in Fig. 15. The binding energies were determined to be − 10.56 kcal/mol. CeO_2_NPs made nine hydrogen bonds with Tyr406, Thr409, Thr413, Arg415, Glu439, and His441 in the activity pocket. In addition, hydrophobic interactions included one carbon-hydrogen bond with His441 (Fig. 15).

**Fig. 16:**
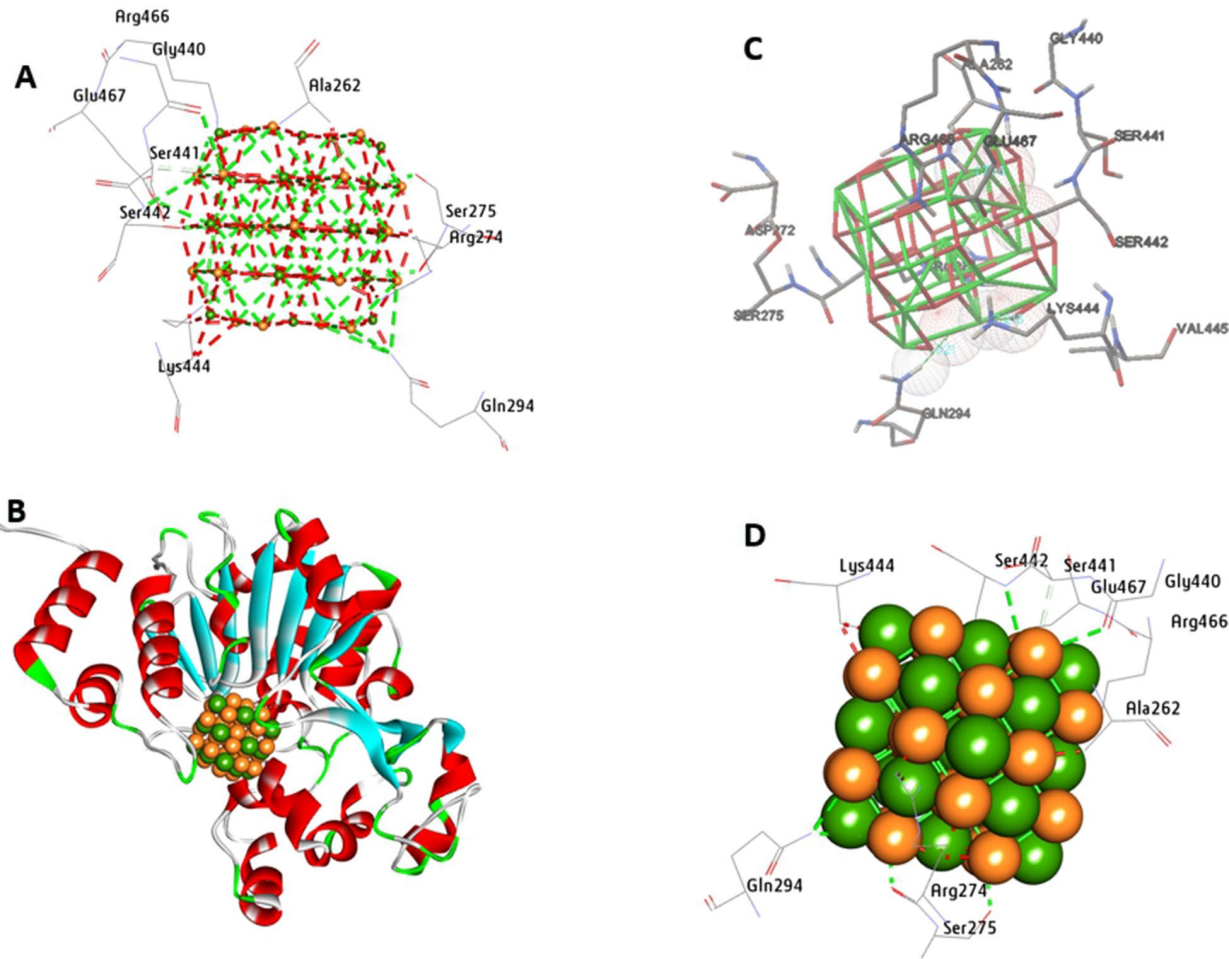
3D depictions of CeO_2_NP conformations within the binding pocket of SIRT 1 (PDB: 5BTR). **A** and **D** display the interaction between shared residues and CeO_2_NPs, **B** complex interaction with CeO_2_NPs, and **C** feature interaction with CeO_2_NPs

The SIRT1, a conserved member of the sirtuin family, enhances DNA repair, mitigates oxidative stress, and suppresses apoptosis, underscoring its importance in cellular resilience. The molecular docking results between CeO_2_NPs and the SIRT1 protein (PDB: 5BTR), revealing a strong binding affinity with a calculated energy of − 11.55 kcal/mol (Fig. 16). Within the activity pocket of SIRT1, CeO_2_NPs formed eight hydrogen bonds with residues Ser275, Gln294, Arg274, Gly440, Ser441, Ser442, Lys444, Arg466, and Glu467. Additionally, a hydrophobic interaction involving a carbon-hydrogen bond was observed with Ser441, further stabilizing the complex (Fig. 16). These interactions highlight the potential structural basis for CeO_2_NPs in modulating SIRT1 activity.

## Discussion

One of the main issues with worldwide public health is liver fibrosis (Sánchez-Valle et al. 2012). Therefore, searching for effective candidates or agents to dampen or reverse the progression of liver fibrosis is highly encouraged. Because TAA-induced hepatic damage in rats closely resembles the morphological and functional changes that are seen in human fibrosis, it is a commonly used animal model of liver fibrosis (Hou et al. 2014; Huang et al. 2014). This article was created to evaluate the influence of eco-friendly plant-based fabricated CeO_2_NPs at 0.1 and 0.5 mg/kg on the main mediators of the fibrotic signaling pathway in a rat animal model of TAA-stimulated liver fibrosis.

TAA caused hepatic injury in our article, with a remarkable elevation in serum GPT, GOT, ALP, and total bilirubin levels and a decline in serum total protein and albumin levels versus the control one, and further confirmed by the histopathological findings. Our data go hand in hand with prior research (Megahed et al. 2023; Radwan et al. 2024). According to Miao et al., TAA could result in cell destruction through obstructing RNA translocation from the nucleus to the cytoplasm, consequently loss of cell function and death, followed by leakage of hepatic enzymes in the serum (Miao et al. 2020). These liver function parameters were retrieved to almost normal values in the CeO_2_NPs-treated rats, which could be correlated with its efficacy in reducing liver tissue damage. These outcomes are in accordance with those of previous reports (Abbasi et al. 2021; Mahmoud 2023).

Chronic liver diseases are mostly accompanied by oxidant/antioxidant imbalance (Uchida et al. 2020). Lipid peroxide products are principal contributors to the stimulation of HSCs in liver fibrosis (Zhao et al. 2021). Overproduction of the byproducts of liver lipid peroxidation may upset the intracellular equilibrium, resulting in oxidative stress, which harms the cell (Horn and Jaiswal 2018). Moreover, inflammatory reaction is considered an essential part in the occurrence of long-standing liver disorders and is usually followed by the onset of liver fibrosis (Zhao et al. 2021). According to Wang et al. (Wang et al. 2013), the intermediate metabolites of TAA contain reactive radicals that cause oxidative stress in hepatocytes, leading to liver damage and lobular center necrosis. Moreover, profibrogenic growth factors, cytokines, and prostaglandins are released in response to ROS generation and products of lipid peroxidation (Crosas-Molist and Fabregat 2015). Nrf2 controls the liver resistance to oxidants (Zhou et al. 2022); it regulates antioxidant-responsive elements expression that, in turn, promotes the production of multiple antioxidant mediators such as HO-1, SOD, and GSH (Abdel‐Rahman et al. 2022). Also, GSH itself has a major position in getting rid of the toxic metabolites, and when its levels are remarkably declined, the liver injury initiates (Abbasi et al. 2021).

Findings of our study revealed that TAA caused remarkable oxidative damage to the liver, recognized by a marked rise in the hepatic MDA level combined with a decline in GSH content and downregulation of the Nrf2/HO-1 pathway when matched to the normal records. Multiple investigations (Yang et al. 2019; Bradosty et al. 2021; ElBaset et al. 2022) are in accordance with us and observed that the etiology of fibrosis included ROS-mediated liver damage. On the other side, intravenous injection of green-synthesized CeO_2_NPs dose-dependently significantly restored the oxidant/antioxidant balance in liver tissue via activated nuclear translocation of Nrf2 and thus inducing HO-1 and GSH production and blocking MDA liberation/synthesis. Multiple studies go hand in hand with us and revealed that these protective influences of CeO_2_NPs were mainly attributed to its powerful free radical inhibition activity through its double oxidation state, which potentiates its scavenging ability and antioxidant-mimetic characters (Abbasi et al. 2021; Ommati et al. 2021; Godugu et al. 2023).

Furthermore, liver fibrosis is the primary pathophysiologic outcome of chronic hepatic damage, as evidenced by increased extracellular matrix (ECM) deposition (Bataller and David 2005; Hernandez-Gea and Friedman 2011). Liver collagen deposition is a cumulative phenomenon that impairs the liver’s capacity to carry out particular catabolic functions. Intracellular mediators called Smads are responsible for transducing TGF-β1 signaling (Heldin et al. 1997). An important part of the fibrotic process is played by TGF-β1 (Green et al. 2015; Zhou et al. 2018). Upon activation of TGF-β1, Smad2 and Smad3 undergo further phosphorylation and combine with additional Smad proteins to create a heteromeric complex. This complex undergoes nuclear translocation and regulates a variety of fibrotic mediators, such as COL1A1, α-SMA, and MMP-2. Therefore, TGF-β1/Smads signaling may be used as a therapeutic intervention to treat liver fibrosis.

Our findings demonstrated that TAA administration significantly upregulated hepatic TGF-β1, COL1A1, MMP-2, p-Smad2, and p-Smad3 content, giving clear evidence of liver fibrosis incidence. Explicitly, TAA caused the production of ROS, leading to the differentiation of HSCs to myofibroblasts, which is followed by the cell proliferation that upgraded the construction of the ECM and the appearance of α-SMA, exacerbating the liver fibrosis (Kadir et al. 2014; Tsuchida and Friedman 2017).

In our research, the immunofluorescence staining showed that the positive expression of α-SMA was remarkably increased in the hepatic tissue of the TAA-intoxicated group, which is in line with previous research (Mi et al. 2019; Zhou et al. 2021). Besides, the staining results with H&E and Picrosirius red exhibited massive deposition of collagen fibers, bridging fibroplasia and pseudolobulation, and vacuolar degeneration of hepatocytes, signifying that TAA-induced severe liver damage and fibrosis. These findings are in agreement with prior studies (Khalil et al. 2021; Zhao et al. 2021). On top of that, there was karyocytomegaly, mitotic figures, apoptosis, portal fibrosis, infiltration of mononuclear inflammatory cells, and hyperplasia of bile ducts with newly-formed bile ductulus. These findings are in line with those mentioned by El Awdan et al. (El Awdan et al. 2019).

Through disruption of TGF-β1/Smads signaling, we observed in our study that green-fabricated CeO_2_NPs significantly reduced TAA-induced liver fibrosis. CeO_2_NPs markedly suppressed the TGF-β1-induced Smad2 and Smad3 phosphorylation in the hepatic tissues, suggesting the anti-fibrotic potential of CeO_2_NPs. Likewise, previous research reported the anti-fibrotic action of CeO_2_NPs (Boey et al. 2021; Abdel-Karim et al. 2024). Additionally, our data revealed that CeO_2_NPs effectively reduced the degenerative effects of TAA in the liver tissue, as confirmed by the histological examinations with H&E, Picrosirius red, and α-SMA immune-expression. These outcomes suggested the anti-fibrotic action of CeO_2_NPs, which are in accordance with numerous studies (Oró et al. 2016; Boey et al. 2021; Abdel-Karim et al. 2024).

Moreover, SIRT1, which belongs to the sirtuin protein family (Michan and Sinclair 2007), is a NAD-dependent protein responsible for the deacetylation of histones and various non-histone proteins. This process is crucial for gene expression, metabolism, apoptosis, autophagy, and aging (Cohen et al. 2004; Haigis and Guarente 2006; Michan and Sinclair 2007; Yamamoto et al. 2007; Chang et al. 2015). It has been recently reported that Nrf2 may act as a downstream target of the SIRT1 signaling, working in conjunction with SIRT1 to regulate antioxidant expression and thereby influence oxidative stress levels (Huang et al. 2013, 2015, 2017; Do et al. 2014; Ding et al. 2016). In particular, evidence suggests that activating SIRT1 can influence the oxidant status of cells by enhancing the Nrf2/Keap1 pathway’s activity, which leads to an increase in the nuclear levels of Nrf2 and a boost in Nrf2 transcriptional activity (Kulkarni et al. 2014). Consequently, SIRT1 may be involved in safeguarding cells from ROS produced during cellular metabolism or arising from external sources. Prior studies on hepatic dysfunction recorded the downregulation in the SIRT1/Nrf2 pathway (Jia et al. 2021; Isaacs-Ten et al. 2022; Abu-Risha et al. 2023). Furthermore, recent research revealed the potential impact of SIRT1 to act as a potent TGF-β blocker through quenching ROS generation and inhibiting TGF-β/Smad2/3 pathway in many diseases (Huang et al. 2017; Liu et al. 2019; Xiong et al. 2023; Yu et al. 2024). Specifically, SIRT1 has been shown to inhibit the TGF-β1/Smad pathway in liver fibrosis models, leading to a reduction in pro-fibrotic markers such as TGF-β, α-SMA, and CoL1A1. This inhibition results in decreased activation of HSCs and reduced epithelial-mesenchymal transition, ultimately slowing the progression of fibrosis (Pan et al. 2022; Adjei‐Mosi et al. 2023; Qiu et al. 2023).

Based on SIRT1’s vital role in combating liver fibrosis through acting as an Nrf2 stimulant and TGF-β blocker, its level in the hepatic tissues has been evaluated in our study. A significant decline in SIRT1 gene level has been detected in the liver tissue of the TAA-hepatotoxic group, explaining the observed inactivation of Nrf2 pathway coupled with a boost in TGF-β/Smad signaling. On the other side, treatment with CeO_2_NPs dose-dependently succeeded in upregulating the expression of the hepatic SIRT1 gene, maximizing its anti-fibrotic potential. Notably, prior studies documented the positive impact of nanoceria on SIRT1 expression and, accordingly, inhibited the TGF-β pathway and stimulated Nrf2 activation, leading to significant repression in tissue damage (Taha et al. 2022; Elsaid et al. 2024).

Furthermore, the liver of TAA-treated rats showed an increase in caspase-3 (marker of apoptosis). TAA or its metabolite harms DNA, RNA, and protein synthesis in hepatocytes, inducing liver metabolic impairment and even hepatocyte apoptosis. Caspase-3 is a sensitive indicator of liver injury that is also linked to hepatic fibrosis (Bantel et al. 2004). It has been reported that the oxidative stress and inflammation in the hepatic cells provoked by TAA toxicity led to hepatocyte death, evidenced by the increased expression of caspase-3 in the TAA-challenged rats (El Awdan et al. 2019). On the other side, CeO_2_NPs at the given doses significantly reduced hepatic caspase-3 expression, indicating anti-apoptotic effects of CeO_2_NPs. This observation agrees with a previous study (Wasef et al. 2021).

In addition, based on the outcomes of molecular docking studies, CeO_2_NPs showed great binding affinities toward the fibrotic mediators; TGF-β1, COL1A1, MMP-2, Smad2, and Smad3, as well as antioxidant response elements; SIRT1, Nrf2, and HO-1 proteins which supported the biochemical and molecular records. Moreover, these results maximize the potential action of CeO_2_NPs as an anti-fibrotic and antioxidant powerful agent.

## Conclusion

The results attained in this report verified the anti-fibrotic action of plant-based synthesized CeO_2_NPs at the two adopted doses: 0.1 and 0.5 mg/kg rat bw, against TAA-provoked liver fibrosis in rats. Of note, this favorable influence of CeO_2_NPs could be due to its noteworthy antioxidant capacity, thus upregulating SIRT1/Nrf2/HO-1 pathway, consequently blocking TGF-β1/Smad2/3 signaling pathway, and as a result, the fibrotic mechanism was dampened. However, further research could be established to evaluate the therapeutic efficacy of CeO_2_NPs against other anti-fibrotic drugs/agents in counteracting hepatic fibrosis. Additionally, these outcomes may pave the way for additional investigations to scrutinize the usefulness of CeO_2_NPs in the clinical setting, in particular, for patients with co-existing liver diseases.

## Limitations of the study

Future studies could delve deeper into the observed anti-fibrotic effect of CeO_2_NPs against liver fibrosis. Among these studies, in vitro experiments using relevant liver cell models (such as hepatocytes or hepatic stellate cells) treated with hepatotoxic agents such as TAA or similar agents could be conducted to provide definitive evidence of the anti-fibrotic potential of CeO_2_NPs. Furthermore, functional modulation of the Nrf2 pathway using specific inhibitors or activators would help clarify whether the protective effects of the CeO_2_NPs are abrogated when this pathway is inhibited or enhanced when artificially activated.

## Supplementary Information

Below is the link to the electronic supplementary material.

ESM 1 (DOCX 315 KB)

## Data Availability

All data generated or analyzed during this study are included in this published article.
