# Supplementary material for: Cerium oxide nanoparticles attenuate hepatic failure via blocking TGF-β/Smads and upregulating Nrf2/HO-1 signaling pathways in liver fibrosis rat model
Source: Naunyn Schmiedebergs Arch Pharmacol. 2025 Jul 18;399(1):745–67. doi: 10.1007/s00210-025-04435-x (PMC12894128; doi:10.1007/s00210-025-04435-x)
Supplement: Supplementary file 1 — (DOCX 315 KB) [file 210_2025_4435_MOESM1_ESM.docx]

**Cerium oxide nanoparticles attenuate hepatic failure via blocking TGF-β/Smads and up-regulating Nrf2/HO-1 signaling pathways in liver fibrosis rat model**

**Noha A. Mowaad^a^, Sara M. Baraka^b*,^ Saber Ibrahim^c, d^, Doaa A. Mansour^e^, Reda M. S. Korany^f^, Ahmed F. El-Sayed ^g,h^, Arwa A. Hassan^i^**

^a^ Narcotics, Ergogenics and Poisons Department, National Research Centre, Giza 12622, Egypt

^b^ Chemistry of Natural Compounds Department, National Research Centre, Giza 12622, Egypt

^c^ Packaging materials Department, National Research Centre, Giza 12622, Egypt

^d^ Nanomaterials investigation lab., Central laboratory network, National Research Centre, Giza 12622, Egypt

^e^ Department of Biochemistry and Chemistry of Nutrition, Faculty of Veterinary Medicine, University of Sadat City, Egypt

^f^ Pathology Department, Faculty of Veterinary Medicine, Cairo University

^g^ Microbial Genetics Department, Biotechnology Research Institute, National Research Centre, Giza 12622, Egypt

^h^ Egypt Center for Research and Regenerative Medicine (ECRRM), Cairo, Egypt

^i^ Pharmacology and Toxicology, Ministry of Health & Population, Egypt

*Corresponding author: Chemistry of Natural Compounds Department, National Research Centre, Giza 12622, Egypt (e-mail: [sm.baraka@nrc.sci.eg](mailto:sm.baraka@nrc.sci.eg)) (ORCiD, 0000-0002-3714-2120)

**Table 1S. List of targets, crystallization method, PDB IDs, resolution, and active site coordinates.**

| **NO** | **Protein Targets** | **Method** | **PDB ID** | **Resolution** | **Active site coordinates:** | | | **References** |
| --- | --- | --- | --- | --- | --- | --- | --- | --- |
|  |  |  |  |  | **X** | **Y** | **Z** |  |
| 1 | Transforming growth factor 1 | **X-RAY** | **2X7O** | 3.70 Å | 89.48 | 59.91 | 58.62 | [1] |
| **2** | Smad3 | **X-RAY** | **1OZJ** | 2.40 Å | 24.46 | -5.85 | 17.77 | [2] |
| **3** | Nuclear factor erythroid 2 | **X-RAY** | **7ECA** | 2.00 Å | 36.90 | 12.04 | 0.558 | [3] |
| **4** | Matrix Metalloproteinase 2 | **X-RAY** | **1CK7** | 2.80 Å | 44.76 | 96.35 | 150.8 | [4] |
| **5** | Collagen 1A1 | **X-RAY** | **5K31** | 2.20 Å | 2.11 | -7.49 | 110.0 | [5] |
| **6** | Heme oxygenase-1 | **X-RAY** | **1N45** | 1.50 Å | 14.70 | 0.734 | -2.20 | [6] |
| **7** | Smad2 | **X-RAY** | **1KHX** | 1.80 Å | 78.13 | 92.75 | 40.62 | [7] |
| **8** | SIRT1 | **X-RAY** | **5BTR** | 3.20 Å | -18.33 | 56.00 | 10.85 | [8] |

**Table 2S.** Molecular interactions of Cerium oxide nanoparticles (CeO_2_NPs) with amino acids of list of targets (amino acids showing similar interactions are marked in bold and red color).

|  | **Ligands** | **Proteins** | **3D Structure** | **Hydrophilic Interactions** | | **Hydrophobic Contacts** | | **No. of**  **H-Bonds** | **No. of**  **Total Bonds** | **affinity**  **kcal mol-1** |
| --- | --- | --- | --- | --- | --- | --- | --- | --- | --- | --- |
|  |  |  |  | **Residue (H- Bond)** | **Length** | **Residue (Bond type)** | **Length** |  |  |  |
| 1 | **CeO_2_NPs** | **Transforming growth factor β1** | **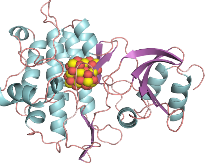** | Tyr249 (H- Bond)  His317 (H- Bond)  His331 (H- Bond)  Arg332 (H- Bond)  Asn338(H- Bond)  Ala350(H- Bond)  Asp400(H- Bond)  Ala350(H- Bond) | 3.07  2.92  1.88  2.78  3.07  1.89  2.72  2.80 | Leu313, (C-H-Bond)  Leu316, (C-H-Bond)  Leu316, (C-H-Bond)  Leu316, (C-H-Bond)  Ile349, (C-H-Bond)  Ile349, (C-H-Bond)  His331(Pi-Sigma)  Ile329, (Alkyl)  Leu352, (Alkyl) | 2.82  3.22  2.92  3.10  3.09  3.74  3.26  4.76  5.27 | **12** | **22** | **-18.55** |
| 2 |  | **Smad3** | **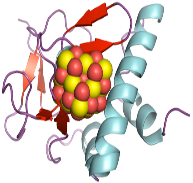** | Trp30 (H- Bond)  Val77 (H- Bond)  Arg80 (H- Bond)  Arg90(H- Bond)  Val86(H- Bond)  Ile87(H- Bond)  Lys81(H- Bond)  Glu27(H- Bond) | 2.80  3.05  3.01  3.09  3.20  2.47  3.09  2.90 | Cys31, (C-H Bond)  Arg80, (C-H Bond)  Gly82, (C-H-Bond)  Trp30, ((C-H-Bond))  Trp30, (C-H-Bond)  Trp30, (Pi-Sigma)  Trp30, (Pi-Sigma) | 3.30  3.22  3.15  2.57  2.41  2.84  3.76 | **10** | **18** | **-** **22.84** |
| 3 |  | **Nuclear erthyroid related factor 2** | 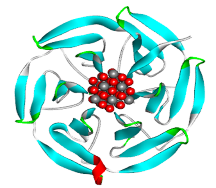 | Val369 (H- Bond)  Val420 (H- Bond)  Val467 (H- Bond)  Val514(H- Bond)  Thr560(H- Bond)  Val561(H- Bond)  Val608(H- Bond)  Val369(H- Bond) | 2.07  2.42  1.84  2.07  2.70  1.76  2.31  2.49 | Val608(C-H bond)  Val369(C-H bond) | 3.55  4.32 | **10** | **12** | **-12.497** |
| 4 |  | **Matrix metalloproteinase2** | 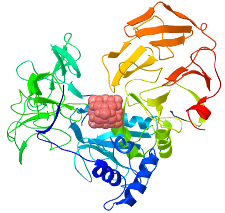 | Gln393, (H-Bond)  Tyr395, (H-Bond)  Ser546, (H-Bond)  Gly394, (H-Bond)  Tyr425, (H-Bond)  Asp392, (H-Bond)  Gln393, (H-Bond)  Ala510, (H-Bond)  Ser546, (H-Bond)  Thr511, (H-Bond) | 3.32  2.49  2.95  2.72  3.30  2.87  3.25  3.17  3.26  2.56 | Pro100, (C-H bond)  Pro100, (C-H bond) | 3.42  3.51 | **12** | **14** | **-** **16.48** |
| 5 |  | **Collagen 1A1** | 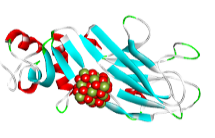 | Gln147, (H-Bond)  Asp239, (H-Bond) | 2.80  2.69 | Gly241, (C-H bond)  Pro242, (C-H bond) | 2.93  3.27 | **2** | **4** | **-6.87** |
| 6 |  | **Heme-oxygenase 1** | 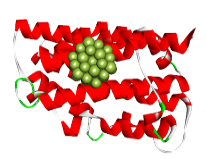 | Gln112, (H-Bond)  His119, (H-Bond)  Gln112, (H-Bond)  Tyr107, (H-Bond) | 2.03  2.73  2.95  2.80 | Lys116, (C-H bond)  His119, Pi-Donor-bond) | 3.31  4.14 | **4** | **6** | **-18.10** |
| 7 |  | **Smad2** | 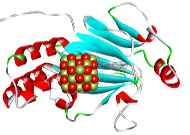 | Tyr406, (H-Bond)  Thr409, (H-Bond)  Thr413, (H-Bond)  Arg415, (H-Bond)  Glu439, (H-Bond)  His441, (H-Bond) | 2.88  2.53  2.66  2.89  3.19  2.93 | His441, (C-H bond) | 2.81 | **9** | **10** | **-10.56** |
| 8 |  | **SIRT1** | 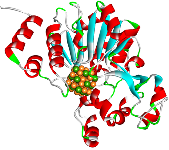 | Ser275, (H-Bond)  Gln294, (H-Bond)  Ser442, (H-Bond)  Lys444, (H-Bond)  Arg466, (H-Bond)  Glu467, (H-Bond)  Gly440, (H-Bond)  Arg274, (H-Bond) | 2.34  2.92  2.97  3.07  2.57  2.78  2.84  2.54 | Ser441, (C-H bond) | 2.84 | **8** | **9** | **-11.55** |

**Ala**: Alanine, **Arg**: Arginine, **Asn:** Asparagine **Asp:** Aspartic acid, **Cys:** Cysteine, **Glu:** Glutamic acid, **Gln:** Glutamine, Gly: Glycine, **His:** Histidine, **Ile:** Isoleucine, **Leu:** Leucine, **Lys:** Lysine, **Met:** Methionine, **Phe:** Phenylalanine, **Pro:** Proline, **Ser:** Serine, **Thr:** Threonine, **Trp:** Tryptophan, **Tyr:** Tyrosine, **Val:** Valine.

References

[1] G.J. Roth, A. Heckel, T. Brandl, M. Grauert, S. Hoerer, J.T. Kley, G. Schnapp, P. Baum, D. Mennerich, A. Schnapp, Design, synthesis, and evaluation of indolinones as inhibitors of the transforming growth factor β receptor I (TGFβRI), J. Med. Chem. 53 (2010) 7287–7295.

[2] J. Chai, J.-W. Wu, N. Yan, J. Massagué, N.P. Pavletich, Y. Shi, Features of a Smad3 MH1-DNA Complex: roles of water and zinc in DNA binding, J. Biol. Chem. 278 (2003) 20327–20331.

[3] L. Cheng, H. Wang, S. Li, Z. Liu, C. Wang, New insights into the mechanism of Keap1-Nrf2 interaction based on cancer-associated mutations, Life Sci. 282 (2021) 119791.

[4] E. Morgunova, A. Tuuttila, U. Bergmann, M. Isupov, Y. Lindqvist, G. Schneider, K. Tryggvason, Structure of human pro-matrix metalloproteinase-2: activation mechanism revealed, Science (80-. ). 284 (1999) 1667–1670.

[5] U. Sharma, L. Carrique, S. Vadon-Le Goff, N. Mariano, R.-N. Georges, F. Delolme, P. Koivunen, J. Myllyharju, C. Moali, N. Aghajari, Structural basis of homo-and heterotrimerization of collagen I, Nat. Commun. 8 (2017) 14671.

[6] L. Lad, D.J. Schuller, H. Shimizu, J. Friedman, H. Li, P.R.O. de Montellano, T.L. Poulos, Comparison of the heme-free and-bound crystal structures of human heme oxygenase-1, J. Biol. Chem. 278 (2003) 7834–7843.

[7] J.-W. Wu, M. Hu, J. Chai, J. Seoane, M. Huse, C. Li, D.J. Rigotti, S. Kyin, T.W. Muir, R. Fairman, Crystal structure of a phosphorylated Smad2: Recognition of phosphoserine by the MH2 domain and insights on Smad function in TGF-β signaling, Mol. Cell. 8 (2001) 1277–1289.

[8] Cao, D., Wang, M., Qiu, X., Liu, D., Jiang, H., Yang, N., & Xu, R. M. (2015). Structural basis for allosteric, substrate-dependent stimulation of SIRT1 activity by resveratrol. *Genes & development*, *29*(12), 1316-1325.‏
